# Treatment Patterns for Incident Bipolar Disorder Among Nonrefugee Immigrants, Refugees, Second‐Generation Immigrants, and Host Population in Sweden

**DOI:** 10.1111/bdi.70007

**Published:** 2025-03-07

**Authors:** Alexander Kautzky, Emma Pettersson, Ridwanul Amin, Aemal Akhtar, Antti Tanskanen, Heidi Taipale, Johannes Wancata, Katalin Gemes, Ellenor Mittendorfer‐Rutz

**Affiliations:** ^1^ Division of Insurance Medicine, Department of Clinical Neuroscience Karolinska Institutet Stockholm Sweden; ^2^ Department for Psychiatry and Psychotherapy Medical University of Vienna Vienna Austria; ^3^ Department of Forensic Psychiatry University of Eastern Finland, Niuvanniemi Hospital Kuopio Finland; ^4^ School of Pharmacy, University of Eastern Finland Kuopio Finland

**Keywords:** antidepressants, antipsychotics, bipolar disorder, lithium, migration, mood stabilizers, refugees

## Abstract

**Background:**

Deviations in treatment practices toward immigrant groups compared to host populations are common in mental disorders but unknown in bipolar disorder (BD). We aim to close this research gap by analyzing age‐stratified use patterns of antidepressants, mood stabilizers, and antipsychotics following an incident diagnosis of BD in Swedish‐born, second‐ and first‐generation nonrefugee immigrants, and refugees.

**Methods:**

Individuals with incident BD between 2006 and 2018 were identified through Swedish national registers. Medication use was followed up until 5 years after diagnosis. Use rates adjusted for sociodemographic and disease‐related covariates were computed with generalized estimation equations for each population group. Marginal means with 95% confidence intervals (CIs) and significance tests for main and interaction effects of population group and time points are presented. Furthermore, significant effects of population group, age group, time point, and their interaction were tested by Type III joint test yielding *F* and *p* values.

**Results:**

Three months after diagnosis, estimated rates of lack of treatment differed significantly between population groups (*p* < 0.0001) as Swedish‐born (17.3%, CI: 16.8–17.7) lacked disease‐specific treatment less often than second‐generation immigrants (21.1%, 19.7–22.5), first‐generation nonrefugee immigrants (23.1%, 21.3–25.0) and refugees (26.8%, 24.4–29.4). Antidepressant monotherapy was estimated in 17.7% (17.2–18.1) of Swedish‐born, 16.8% (15.5–18.3) of second‐generation immigrants, 17.7% (16.2–19.4) of first‐generation nonrefugee immigrants, and was most prevalent in refugees (20.3%, 18.2–22.7; population group *p* = 0.0002). Mood stabilizers were most dispensed by Swedish‐born (51.3%, 50.6–51.9), followed by second‐generation (47.9%, 46.1–49.8) and first‐generation nonrefugee immigrants (44.5%, 42.4–46.7) and refugees (35.4%, 32.8–38.2; population group *p* < 0.0001). Use rates of antipsychotics were similar between population groups (*p* > 0.05) and estimated at 14.1% (13.7–14.6) in Swedish‐born, 14.0% (12.8–15.3) in second‐generation, 13.0% in first‐generation nonrefugee immigrants (12.0–14.6), and 12.9% (11.1–15.0) in refugees. Following up significant interactions of population and age group, lithium use was estimated to be lower in refugees aged 36–65 years (9.9%, 7.9–12.5; population group *p* = 0.0008) and olanzapine use to be higher in refugees aged 16–35 (9.2%, 7.1–11.9; population group *p* = 0.0002), respectively, compared to other population groups of the same age.

**Conclusions:**

Immigrants, especially refugees, are at risk of not receiving adequate treatment following BD diagnosis, putatively owing to a lack of transcultural competence in healthcare, economic restraints, and community factors. Antidepressant monotherapy should be reduced, while recommended options such as mood stabilizers and specifically lithium should be considered more often.

## 1 Introduction

Immigrants, and especially refugees, are oftentimes exposed to traumatic experiences that put them at risk of mental health disorders such as posttraumatic stress disorder [1], major depression [2], and schizophrenia [3]. Difficulties postmigration include racism, limited access to the labor market, and adjustment to new environments, impacting in different ways refugee and nonrefugee immigrants as well as their offspring born in the new country of residence [4, 5]. Considering that migration is becoming more frequent worldwide and will affect 20% of the Swedish population in 2023 [6], psychiatric care for immigrant groups constitutes an urgent challenge for the healthcare system [7].

Characterized by switching states of depression and mania, bipolar disorder (BD) is a severe mood disorder estimated to affect 2% of the population and to reduce potential lifetime by 10–20 years [8]. Recommended treatments include atypical antipsychotics and mood stabilizers, potentially accompanied by antidepressants [8]. Lithium is considered the gold standard for maintenance treatment but can also be used in acute episodes [9]. This intricate prescription pattern of different drug classes complicates guideline‐conform treatment [10], and both low rates of lithium and high rates of antidepressant monotherapy are concerning [10, 11, 12, 13, 14].

Seminal studies on clinical practice in Northern countries comparing host populations and immigrant groups with psychosis spectrum disorders and major depressive disorder reported a reduced likelihood for immigrants to receive adequate treatment while discontinuation rates were higher [15, 16, 17, 18]. Explanations include a lack of transcultural competence in the healthcare systems, economic marginalization, and cultural aspects such as a preference for treatment outside of academic medicine in immigrants [19, 20, 21]. Comparable studies on BD are lacking, and regarding Sweden, treatment practices for BD have yet to be analyzed on a population level. To bridge these knowledge gaps, treatment patterns among a Swedish national cohort of individuals with an incident diagnosis of BD with regard to first‐ and second‐generation immigration backgrounds and refugee status were analyzed longitudinally and adjusted for important covariates such as education, sickness absence, and disability pension.

## 2 Methods

### 2.1 Study Population

Individual‐level de‐identified data were linked from national registers [22, 23, 24, 25, 26, 27, 28], listed in Table S1, to select all individuals with an incident diagnosis of BD aged 16–65 years and registered in Sweden 07/01/2006–09/31/2018.

Diagnoses documented according to International Classification of Diseases version 10 (ICD‐10) Codes F30 and F31 in inpatient or specialized outpatient healthcare, that is, secondary healthcare, and in sick leaves or disability pensions were considered. Individuals were required to have lived 3 years in Sweden to ensure a reasonable time frame of exposure to the Swedish healthcare system. Furthermore, only individuals without use of antipsychotics or mood stabilizers within 15–3 months prior to the diagnosis of BD were considered. This time window was implemented on one hand to restrict the analysis to individuals that had not been treated with medication typically used for BD in the year prior to diagnosis, thus excluding patients that may not be true incident cases. On the other hand, we implemented a 3‐month tolerance for medication use preceding diagnosis as in clinical care oftentimes treatment is initiated before a formal diagnosis is given. Subjects with a diagnosis of psychosis spectrum disorders (ICD‐10: F20–29) or dementia (ICD‐10: F00‐03, G30) registered in secondary healthcare within 3 years prior were excluded.

Time trends in drug use patterns were captured by splitting the study sample into three groups based on diagnosis year: 2006–2009, 2010–2013, and 2014–2018.

### 2.2 Population Groups

Individuals with BD were grouped into (1) Swedish‐born: individuals as well as both their parents born in Sweden, including individuals with missing information on one parent; (2) second‐generation immigrants: born in Sweden but with at least one parent born outside the Nordic countries; (3) first‐generation nonrefugee immigrants: born outside Sweden without refugee status; and (4) refugees: immigrants with refugee status granted by the Swedish migration agency. To ensure comparability within immigrant groups, second‐generation immigrants born to two parents from Nordic countries and nonrefugee immigrants with countries of birth not registered within the refugee group were excluded. For countries of birth and average duration of residence in Sweden among first‐generation immigrants, see Table S2.

### 2.3 Medication

Drug use periods were modeled with PRE2DUP based on dispensing dates [29]. Drugs were identified by Anatomical Therapeutic Chemical classification system codes (ATC) and grouped as antidepressants, mood stabilizers, oral antipsychotics, and sedatives as detailed in Table S3. Furthermore, the lack of pharmacological treatment for BD (neither antidepressants, antipsychotics, nor mood stabilizers), antidepressant monotherapy (lack of concomitant treatment with mood stabilizers or antipsychotics), and long‐acting injectable (LAI) formulations of antipsychotics were assessed. The most common antidepressant and antipsychotic agents not analyzed on a drug level are listed in Table S4.

### 2.4 Covariates

Socio‐demographic variables, including sex, age, educational level, family situation, sickness absence, and disability pension, were assessed 1 year prior to diagnosis. Furthermore, mental and somatic comorbidities diagnosed in secondary healthcare as main or side diagnoses defined by ICD‐10 codes were assessed cross‐sectionally for the 3 years prior to BD diagnosis. Table 1 presents a detailed characterization of covariates.

**TABLE 1:** Baseline characteristics of the study population with incident diagnosis of bipolar disorder (2006–2018) in Sweden, grouped by migration background.

Characteristic^a^	Swedish‐born	Second‐generation immigrants^b^	First‐generation immigrants	
			Nonrefugees	Refugees
	26,931 (81%)	2927 (8.9%)	2013 (6.1%)	1182 (3.6%)
Sex, *n* (%)				
Women	17,086 (63.4)	1874 (64.0)	1275 (63.3)	712 (60.2)
Men	9845 (36.6)	1053 (36.0)	738 (36.7)	470 (39.8)
Age				
Mean (SD)	35.6 (12.8)	32.0 (12.3)	38.2 (11.9)	37.9 (11.2)
16–35, *n* (%)	14,683 (54.5)	1979 (67.6)	922 (45.8)	532 (45.0)
Education years, *n* (%)				
Missing	399 (1.5)	82 (2.8)	68 (3.4)	24 (2.0)
0–9	6011 (22.3)	770 (26.3)	403 (20.0)	281 (23.8)
10–12	12,748 (47.3)	1263 (43.1)	821 (40.8)	450 (38.1)
> 12	7773 (28.9)	812 (27.7)	721 (35.8)	427 (36.1)
Family situation, *n* (%)				
Single, without children	16,774 (62.3)	2102 (71.8)	1108 (55.0)	610 (51.6)
Single, with children	2546 (9.5)	230 (7.9)	190 (9.4)	133 (11.3)
Cohabitant, without children	2145 (8.0)	155 (5.3)	220 (10.9)	124 (10.5)
Cohabitant, with children	5466 (20.3)	440 (15.0)	495 (24.6)	315 (26.6)
Sickness absence in days, *n* (%)				
0	16,949 (62.9)	2018 (68.9)	1337 (66.4)	748 (63.3)
1–90	2851 (10.6)	251 (8.6)	187 (9.3)	112 (9.5)
91–365	7131 (26.5)	658 (22.5)	489 (24.3)	322 (27.2)
Disability pension, *n* (%)				
No	23,924 (88.8)	2676 (91.4)	1770 (87.9)	1076 (91.0)
Yes	3007 (11.2)	251 (8.6)	243 (12.1)	106 (9.0)
Comorbidities, *n* (%)				
Substance use	3464 (12.9)	345 (11.8)	215 (10.7)	111 (9.4)
Depression	10,845 (40.3)	1156 (39.5)	766 (38.1)	495 (41.9)
Anxiety	7619 (28.3)	854 (29.2)	471 (23.4)	291 (24.6)
PTSD	475 (1.8)	98 (3.3)	71 (3.5)	108 (9.1)
Nervous system disorder	2195 (8.2)	245 (8.4)	177 (8.8)	109 (9.2)
Cancer	1716 (6.4)	171 (5.8)	152 (7.6)	79 (6.7)
Circulatory system disorder	1718 (6.4)	125 (4.3)	138 (6.9)	83 (7.0)
Musculoskeletal disorder	4264 (15.8)	432 (14.8)	393 (19.5)	223 (18.9)
Obesity/diabetes	854 (3.2)	95 (3.2)	71 (3.5)	52 (4.4)
History of suicide attempts	1808 (6.7)	201 (6.9)	122 (6.1)	59 (5.0)
Year of diagnosis, *n* (%)				
2006–2009	7137 (26.5)	1253 (42.8)	565 (28.1)	262 (22.2)
2010–2013	9184 (34.1)	978 (33.4)	660 (32.8)	430 (36.4)
2014–2018	10,610 (39.4)	696 (23.8)	788 (39.1)	490 (41.5)
Region of birth				
European Union	26,931 (100)	2947 (100)	788 (39.1)	490 (41.5)
Europe, not European Union			660 (32.8)	430 (36.4)
Outside Europe			565 (28.1)	262 (22.2)

Abbreviations: PTSD, posttraumatic stress disorder; SD, standard deviation.

^a^ Characteristics for sociodemographic factors, sickness absence, and disability pension were assessed cross‐sectionally for a time period of 1 year prior to diagnosis. Comorbidities were assessed cross‐sectionally for 3 years prior to diagnosis.

^b^ The group of second‐generation immigrants was born in Sweden; however, to parents with personal migration history.

### 2.5 Statistical Analyses

Follow‐up started at first BD diagnoses and ended after 5 years or at censoring due to death, emigration, diagnosis of psychosis spectrum disorder or dementia, or end of data linkage (31/12/2018). Use of specific psychopharmacological agents was assessed every 3 months after BD diagnosis for the first year and every 6 months onward to 5 years. For each time point, prevalence was measured over a 14‐day time window as use versus nonuse. Patients with ≥ 10 days in inpatient care were censored for this specific time point as inpatient medication use was not available. Prevalence rates (marginal means) with 95% confidence intervals (CIs) were estimated by Log‐Poisson generalized estimating equation (GEE) models as provided by “R” packages “geepack” [30] and “emmeans” [31], applying robust sandwich standard errors and specifying an AR [1] correlation structure. Separate models were computed for each outcome variable (binomial, using vs. not using) with main effects of time point (factor, 12 levels), population group (factor, four levels), age group (binomial, 16–35 vs. 36–65 years), as well as their interactions. All models were adjusted for socio‐demographic covariates, comorbidities, sickness absence, and disability pension. Effects were tested by Type‐III joint tests and reported by *F* values with degrees of freedom. Whenever insignificant, interactions between age and population groups were dropped from the model. In the case of significant interactions, subjects were dichotomized by age groups of 16–35 and 36–65 years, and post hoc GEE models for main effects of time point and population group, as well as their interaction, were computed. Finally, the effects of covariates on lack of adequate treatment, antidepressant, mood stabilizer, and antipsychotic use were visualized across the whole sample as a forest plot of relative risk (RR) with CI.

For analysis of the time period of diagnosis, follow‐up was reduced to 3 years considering that patients diagnosed in 2014–2018 could not be followed up for 5 years. GEE models were computed for outcomes of no treatment, use of antidepressants, antipsychotics, mood stabilizers, and lithium, including main effects of time point (factor, eight levels), time period of diagnosis (factor, three levels), and their interaction.

Data management and statistical analyses were performed with R 4.2.2 and SAS 9.4.

## 3 Results

The final study population included 33,053 subjects detailed in Table 1. A list of estimated marginal means with 95% CIs for each outcome and population group can be found in Table S5. For a complete list of joint test results, please refer to Table S6.

**FIGURE 1:**
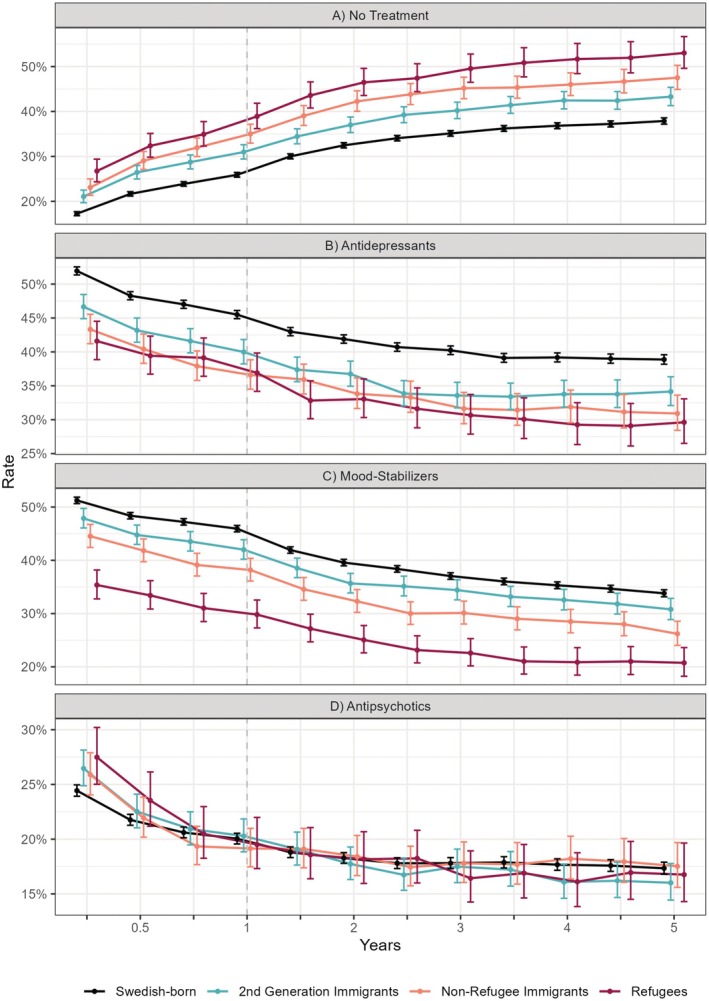
Marginal means with corresponding 95% confidence intervals of estimated rates of (A) no treatment, and use of drug classes (B) antidepressants, (C) mood stabilizers, and (D) oral antipsychotics. Rates are adjusted for socio‐demographic and comorbidity covariates and pictured over follow‐up of 5 years after diagnosis of bipolar disorder. Colored lines correspond to groups based on history of immigration. The group of second‐generation immigrants was born in Sweden, however, to parents with personal migration history.

Lack of pharmacological treatment for BD was estimated for 26.8% of refugees, 23.1% of nonrefugee immigrants, 21.1% of second‐generation immigrants, and 17.3% of Swedish‐born (Figure 1A). The proportion of untreated patients continuously increased across the follow‐up in all groups to a maximum of 53.0% estimated in refugees, indicating higher rates of discontinuation among immigrant groups.

### 3.1 Antidepressants

**FIGURE 2:**
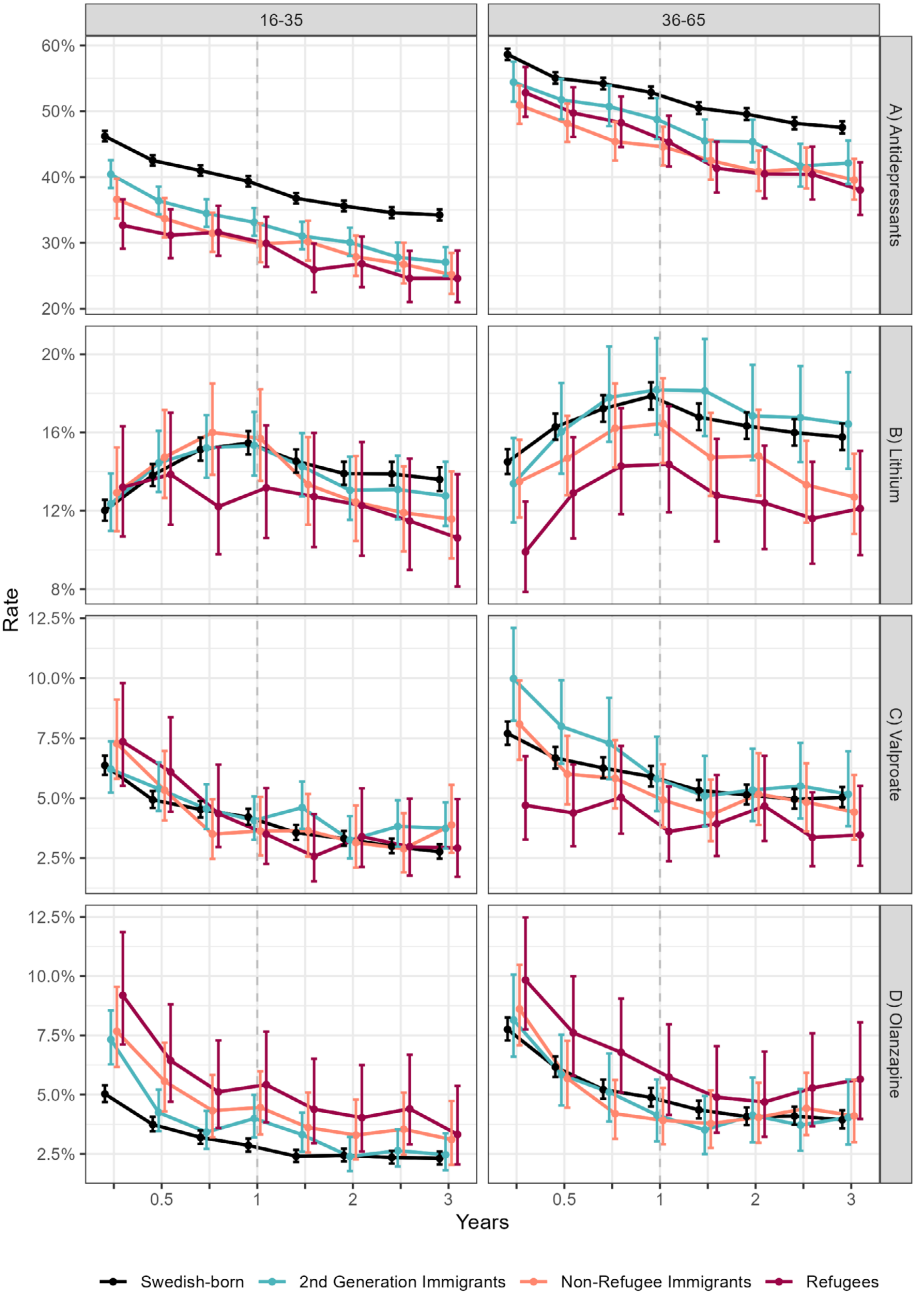
Respectively for patients aged 16–35 and 36–65, marginal means with corresponding 95% confidence intervals of estimated use of (A) antidepressants, (B) lithium, (C) valproate, and (D) olanzapine. Rates are adjusted for socio‐demographic and comorbidity covariates and pictured over a follow‐up of 3 years after diagnosis. Colored lines correspond to groups based on history of immigration. The group of second‐generation immigrants was born in Sweden, however, to parents with personal migration history.

Antidepressants were the most common treatment of BD (Figure 1B). An interaction effect between population and age groups was observed (*F* ^1,3^ = 4.6, *p* = 0.003). Use rates 3 months after diagnosis were 52.9% in refugees and 58.6% in Swedish‐born individuals aged 36–65. A stronger contrast was present in patients aged 16–35 years, with rates of 32.7% observed in refugees compared to 46.2% in Swedish‐born individuals. While usage declined continuously over time in all groups, the rates were consistently higher in Swedish‐born individuals. Figure 2A and Table S7 provide marginal means with CI computed for population groups stratified by age.

**FIGURE 3:**
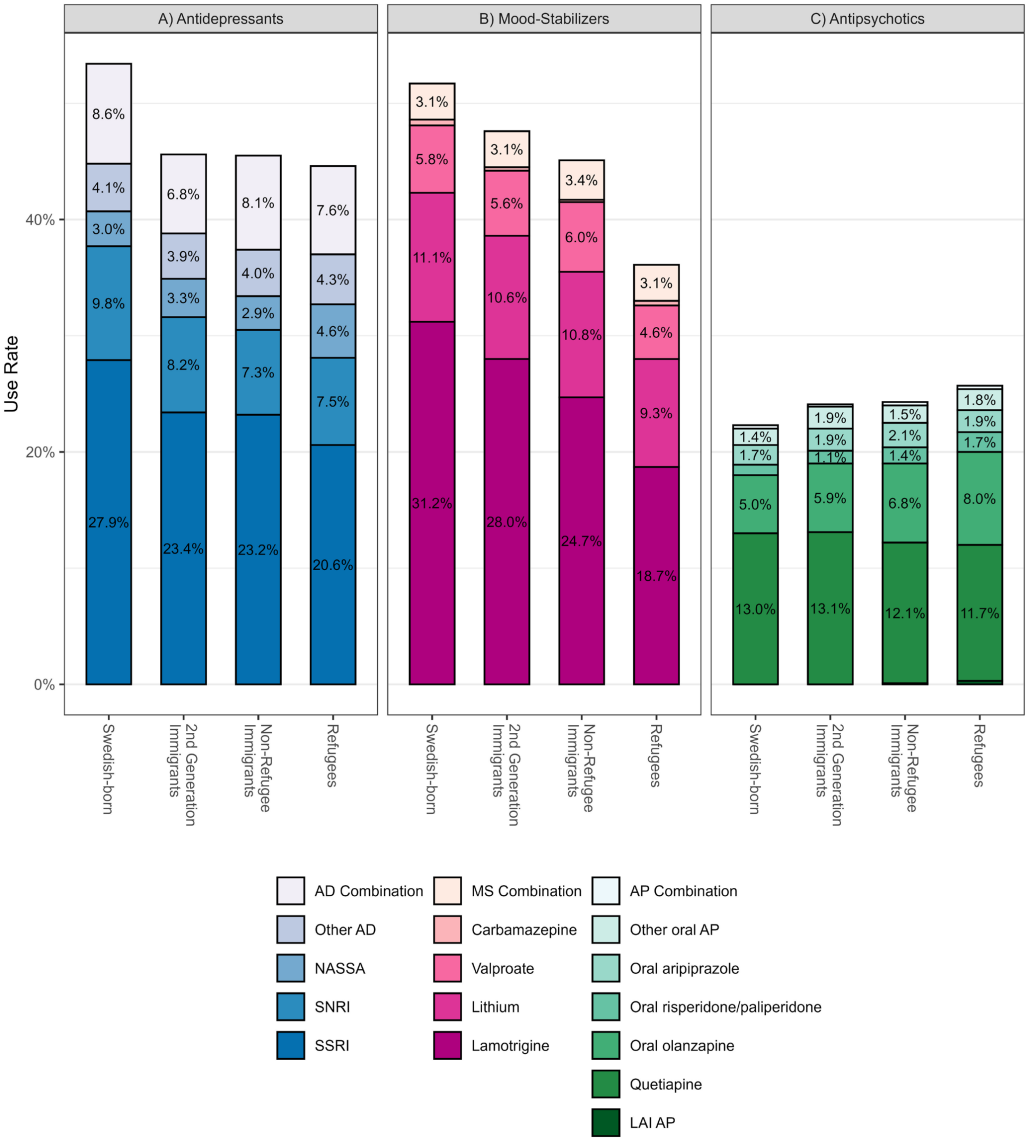
Bar plots depicting the composition of drug use by subclasses and specific drugs used 3 months after incident diagnosis of bipolar disorder. Drugs are grouped as (A) antidepressants, (B) mood stabilizers, and (C) oral antipsychotics, and bar plots are shown respectively for groups based on history of immigration. Crude rates of use are shown (without adjustment for socio‐demographic and comorbidity covariates). Abbreviations: AD, antidepressant; AP, antipsychotic; LAI, long‐acting injectable; MS, mood stabilizer; NASSA, noradrenergic and specific serotonergic antidepressant (mirtazapine; SNRI, serotonin–noradrenaline reuptake inhibitor; SSRI, selective serotonin reuptake inhibitor. Footnote: The group of second‐generation immigrants was born in Sweden, however, to parents with personal migration history.

Bar plots showing composite medication use by subclasses 3 months after diagnosis for all drug classes are provided in Figure 3. Prevalence of antidepressant subclasses is shown in Table S1. Antidepressant monotherapy was observed in 17.7% of all incident BD subjects and was more common in refugees (20.3%) compared to other groups. Rates remained stable across follow‐up.

Three months after diagnosis, SSRI and SNRI were used by 33.3% and 11.9% of Swedish‐born individuals compared to 25.2% and 8.7% of refugees. Use declined over the observation period in all groups for both SSRI and SNRI. Mirtazapine was the third most prevalent antidepressant and was constantly more common in refugees (7.9%, 3 months after diagnosis) compared to other groups. Rates were declining over time in all groups.

### 3.2 Mood Stabilizers

Mood stabilizers were used by 51.3% of Swedish‐born, 47.9% of second‐generation immigrants, 44.5% of nonrefugee immigrants, and 35.4% of refugees 3 months after diagnosis (Figure 1C). Adjusted use rates declined over time while maintaining differences between groups.

Prevalence of mood stabilizer subclasses is shown in Table S2. The pattern regarding the most prevalent agent lamotrigine mimicked overall mood stabilizer use rates. The highest use of 32.6% was estimated 3 months after diagnosis in Swedish‐born individuals, compared to the lowest rates of 20.2% in refugees. In contrast, three‐way interactions between time point, population, and age groups were computed respectively for the second and third most common mood stabilizers, lithium (*F* ^1,33^ = 1.8, *p* = 0.004) and valproate *F* ^1,33^ = 1.8, *p* = 0.005). Among younger patients, lithium (12.7%, Figure 2B) and valproate (7.0%, Figure 2C) use rates were comparable across groups 3 months after diagnosis. However, divergent patterns were estimated in patients aged 36–65 years. Lithium showed lower rates in refugees (9.9%) compared to other groups, especially Swedish‐born patients (14.5%). Valproate use was also estimated to be the lowest in refugees (4.7%) and rates ranged among other groups from 7.7% in Swedish‐born individuals to 10.0% in second‐generation immigrants. Lithium was the only drug peaking in use 1 year after diagnosis across all groups. No effects of population group were observed for carbamazepine and oxcarbazepine, which were used in less than 1% of subjects with BD.

### 3.3 Antipsychotics

Antipsychotics were the least common drug class used in incident BD in Sweden, and no significant differences between groups were found (Figure 1D). Adjusted rates peaked 3 months after diagnosis at 24.8% across all groups and continuously declined thereafter.

Prevalence of antipsychotic subclasses is shown in Table S3. The most common antipsychotics, quetiapine and olanzapine, also decreased in use throughout follow‐up. Three months after diagnosis, adjusted quetiapine use ranged from 12.9% to 14.1% and was marginally lower both in refugees and nonrefugee immigrants compared to Swedish‐born and second‐generation immigrants.

An interaction effect between population and age group on olanzapine use was computed (*F* ^1,3^ = 3.3, *p* = 0.021). Three months after diagnosis, olanzapine use was estimated at 5.9% compared to 7.6% of patients aged 16–35 and 36–65 (Figure 2D). In younger patients, more frequent use of olanzapine was estimated in refugees (9.2%) compared to Swedish‐born patients (5.0%). No effect of population group was present in older patients. Uncommon treatment choices in Sweden included both aripiprazole (overall 2.2%) and risperidone (overall 1.2%). More frequent use of risperidone was estimated in immigrant groups compared to Swedish‐born patients, while no group effects were present for aripiprazole use. Finally, LAI was rarely used (< 0.4%) and could not be analyzed.

### 3.4 Treatment With Mood Stabilizers and/or Antipsychotics

Three months after diagnosis, use of any guideline‐conform treatment with either mood stabilizers, antipsychotics, or a combination thereof was estimated in 62.5% of Swedish‐born, 59.6% of second‐generation immigrants, 56.1% of nonrefugee immigrants, and 49.9% of refugees (Table S6). While use rates declined over time among all groups, across follow‐up, the gap between Swedish‐born (33.8% after 3 years) and refugees (21.9% after 3 years) widened due to a particularly strong decline in use rates estimated in the refugee group (interaction population group and time point: *F* ^1,3^ = 1.5, *p* = 0.038).

### 3.5 Sedatives

Adjusted use rates of sedatives 3 months after diagnosis ranged from 26.7% in refugees to 29.3% in Swedish‐born and declined across the follow‐up among all groups (Table S4). Group differences were primarily driven by fewer uses of benzodiazepines and, to a lesser degree, z‐drugs among refugees and nonrefugee immigrants compared to Swedish‐born and second‐generation immigrants. No significant effect of group was estimated for propiomazine.

### 3.6 Effects of Covariates

The effects of each covariate on medication use—respectively adjusted for all other covariates—are presented in Table S7. Low education level of 0–9 years as well as living single showed the strongest positive association with lack of adequate treatment, while high education level of > 12 years showed the strongest positive association with mood‐stabilizer use. Having had a prolonged sickness absence > 90 days prior to the diagnosis of BD as well as a comorbid anxiety disorder most increased RR for the use of antipsychotics. Finally, a previous diagnosis of depression showed the strongest positive association with antidepressant use. RR with CI is listed in Table S8.

### 3.7 Time Period of Diagnosis

**FIGURE 4:**
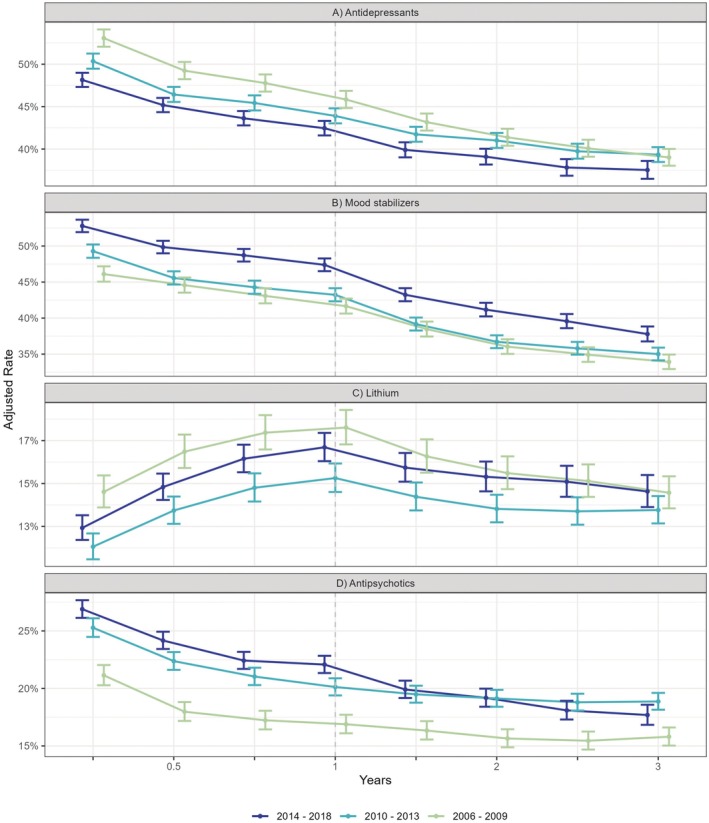
Marginal means of estimated drug use with corresponding 95% confidence intervals stratified by time period of incident diagnosis of bipolar disorder: (A) antidepressants, (B) mood stabilizers, (C) lithium, and (D) oral antipsychotics. Rates are adjusted for socio‐demographic and comorbidity covariates and are pictured over a follow‐up of 3 years after diagnosis. Colored lines correspond to the three cohorts diagnosed in 2006–2009, 2010–2013, and 2014–2018.

Antidepressant use estimated for the time point of 3 months after diagnosis decreased from 53.1% in subjects diagnosed in 2006–2009 to 48.1% in those diagnosed in 2014–2018. Lithium use declined from 14.6% in subjects diagnosed in 2006–2009 to 12.9% in those diagnosed in 2014–2018, while a reverse pattern of increased use was observed for both other mood stabilizers (46.1%–52.8%) and antipsychotics (21.2%–26.9%). For adjusted use rates stratified by time period of diagnosis, please refer to Figure 4 and Table S9.

Use rates of subclasses and specific drugs are detailed in Table S5. Increased use of mood stabilizers and antipsychotics among patients diagnosed between 2014 and 2018 compared to those diagnosed in 2006–2009 was predominantly driven by lamotrigine and quetiapine. Despite relatively stronger increases in lamotrigine use among refugees (9.8% in 2006–2009 to 22.2% in 2014–2018) and nonrefugee immigrants (16.8%–28.8%), overall higher rates were maintained in Swedish‐born (22.7%–37.1%) and second‐generation immigrants (19.8%–32.4%). In contrast, the gap in quetiapine use separating nonrefugee immigrants (5.9%–15.5%) and refugees (4.3%–15.9%) from Swedish‐born (6.7%–16.0%) and second‐generation immigrants (8.5%–14.2%) observed in patients diagnosed in 2006–2009 was not found in those diagnosed between 2014 and 2018.

## 4 Discussion

In this nationwide study on treatment patterns following incident BD diagnosis in Sweden, striking gaps between the Swedish‐born host population and immigrant groups were identified for the use of any targeted treatment, specifically mood stabilizers. More than one‐quarter of refugees compared to less than one in six Swedish‐born patients did not use any treatment for BD 3 months after diagnosis. Only half of refugee patients diagnosed with BD received any guideline‐conform treatment with either mood stabilizers or antipsychotics. Particularly, mood stabilizers were less prevalent in immigrant groups, with adjusted use rates down to 35.4% compared to 51.3% in Swedish‐born individuals, agreeing with the only previous study on differences in medication use between native‐ and foreign‐born patients with BD based on Canadian self‐reported survey data [32]. Prescribers may face difficulties in communicating relevant risks and side effects, but also negative stereotypes toward people with a history of immigration, considering that lower rates were also observed in the treatment of second‐generation immigrants. Insufficient transcultural competence among healthcare professionals and language barriers are known contributors to consistently observed lower contact with mental healthcare in immigrant groups despite similar legislative access to all population groups in Sweden [2, 21]. Gaps may be further widened by out‐of‐pocket costs for adequate treatment that are burdensome for migrant groups, despite a subsidized healthcare system in Sweden [19]. Cultural differences in the perception of mental health leading to alternative treatment practices, such as higher rates of herbal medication, may also compete with psychopharmacological treatment in immigrant groups [20].

Regarding drug classes, incident BD was most often treated with antidepressants both in Swedish‐born and immigrant groups. Mirtazapine was more common in refugees than in other population groups, and 18.6% of refugees compared to 12.6% of Swedish‐born individuals taking antidepressants were using mirtazapine. A similar pattern was observed previously in unipolar depression [16]. More concerning, antidepressant monotherapy is alarmingly common in Sweden, especially in refugees, with the highest rates of 20.3%. Similar findings of overprescribed antidepressants in the treatment of BD have been reported for most countries where population‐wide data are available, including Denmark [11], the United Kingdom [13], Finland [33], and Taiwan [12], as well as by representative surveys for long‐term trends in the United States [34] and Germany [35]. Reasons may include good tolerability and less stigmatization compared to mood stabilizers and antipsychotics, as well as unawareness of poor efficacy and risk of mood switching in BD. On the other hand, widely recognized guidelines such as NICE and derived Swedish national guidelines recommend first‐line combination treatment including antidepressants for bipolar depression, while explicit warnings about antidepressant monotherapy are often lacking [36, 37]. Nevertheless, growing awareness of the shortcomings of antidepressant treatment in BD is suggested by declining use in subjects diagnosed in recent years.

However, the same trend was observed for lithium which was used by 9.3%–11.2% of patients diagnosed in 2014–2018. Declining rates align with international trends, and lower rates of roughly 5% of incident cases treated with lithium were recently shown in Finland [33]. While the notoriety of toxicity and severe long‐term effects such as renal dysfunction may overshadow the abundance of reassuring findings [9, 38], dwindling lithium use may be owed to the overall confusing picture of international treatment guidelines and a failure to provide a definitive recommendation [10]. However, the rapid decline of lithium treatment to 16% of incident cases in 2018 was also observed in the United Kingdom despite NICE guidelines being well‐recognized by UK healthcare and explicitly naming lithium as a first‐line option for long‐term treatment [13, 36]. The distinction between first‐line treatment for acute phases of mania and bipolar depression as well as long‐term treatment may be overly confusing for practical implementation. Despite lithium being the only drug with increasing rates following the diagnosis of BD, the actual numbers of patients who switched to lithium after the acute phase of BD were negligible in Sweden and do not reflect recommendations.

Regarding antipsychotics, use rates remained lower compared to antidepressants and mood stabilizers in Sweden despite increases in quetiapine which accounted for nearly half of the antipsychotics used in all population groups. Findings mirror trends in neighboring countries, Finland and Denmark [11, 33], but contrast with reports from Taiwan and Hong Kong, where antipsychotics rose to the most prescribed drug class in BD treatment observed in more than 70% of cases [12, 13]. Concerning population groups, olanzapine and, to a lesser degree, risperidone were significantly more common in refugees, while aripiprazole and quetiapine were used less often compared to both second‐generation immigrants and Swedish‐born patients. While all four agents are recommended for the treatment of BD, olanzapine and risperidone bring along a higher risk of sedation as well as metabolic side effects. Olanzapine is recommended for acute phases of BD [36]; however, it lacks long‐term tolerability due to pronounced metabolic side effects, including insulin resistance and peripheral inflammation [39]. Considering the highest use of both mirtazapine and olanzapine in refugees, clinicians treating BD may aim for stronger sedation compared to nonrefugee patients, either due to stereotypes or actual differences in symptom presentation, such as higher rates of agitation suggested in refugees by a symptom network analysis [40] or obstacles in gathering sufficient clinical information. Benzodiazepines and z‐drugs commonly used to treat agitation and insomnia were observed less often in refugees here as well as in a Finnish study [15], indicating that concerns about communication difficulties and compliance required for the prescription of addictive drugs may explain this prescription pattern in refugees.

Among the drugs with increasing usage, lamotrigine and quetiapine stick out, respectively, with almost tripled and doubled adjusted use rates in patients diagnosed in 2014–2018 compared to 2006–2009 across all population groups. Quetiapine arguably has the highest versatility among first‐line treatment options for BD, being effective against manic, depressive, and mixed states according to several international guidelines [36, 41, 42, 43]. Despite also being listed among first‐line recommendations by several treatment guidelines [43, 44], the well‐tolerated aripiprazole did not gain comparable traction and was only used by roughly 2.4% of nonrefugee and 1.9% of refugee patients diagnosed in 2014–2018. Lamotrigine, on the other hand, is attributed with the least concern about side effects among drugs named by the Swedish national guidelines for the treatment of BD [37]. Together with depressive states oftentimes being protracted and subjectively more debilitating, this may have promoted the rise of lamotrigine despite lacking efficacy in mania. Interestingly, use rates observed here on the Swedish population level are much lower regarding lithium but higher regarding lamotrigine and antidepressants compared to previous findings in bipolar patients treated in various outpatient facilities across Sweden [45, 46, 47]. Differences may be owed to patient selection and restriction to incident BD, resulting in younger patients that more often receive newer drugs such as lamotrigine and quetiapine rather than lithium [48].

The primary strength of our study is the expansive Swedish national registers that cover all patients treated in Swedish in‐ and specialized outpatient facilities longitudinally, providing validity over a broad spectrum of covariates and minimal loss of follow‐up. Diagnoses of patients only in contact with primary care and private psychiatrists were indirectly compiled from sick leaves and disability pensions; however, most patients with BD can be expected to be diagnosed and treated in specialized care. An important consideration is that immigrants with BD are less likely to get in contact with healthcare and hence to be identified in insurance data registers. This is indirectly supported by the higher proportion of high education levels found in the refugee group. On a speculative note, low socioeconomic status may not only increase the risk of lacking adequate treatment across all groups but also bring along a selection bias, as particularly marginalized groups such as refugees with low education levels may be at risk of not receiving adequate diagnosis of BD. Furthermore, the applied method PRE2DUP is based on dispensing rather than prescription information [29]. Therefore, PRE2DUP pictures actual drug use by patients more closely than other available methods. Regarding limitations, we could not account for gaps between prescription and dispensing that may also show patterns relevant to population groups. Economic marginalization and cultural aspects could contribute to lower rates of adherence to prescribed therapies among immigrants [19]. Furthermore, medication used during hospital stays could not be accounted for, and adequacy of drug dosing was not considered. Also, the analyzed time windows of 14 days are too short to evaluate adequate time for treatment. Any dispensing of medication was considered, meaning that some patients identified as medication users may only have picked up their medication once. Furthermore, the definition of incident BD may be compromised by migrants having received diagnoses prior to emigrating from their home country. While 3 years of residency were required to counteract this issue, gaps in recognition of former BD diagnosis and treatment initiation by Swedish healthcare may exceed 3 years. Finally, interpretation of results is limited by the nature of register‐based studies that do not allow symptoms and disease severity to be considered.

## 5 Conclusion

In summary, Swedish treatment practices for BD call for reassessment of poor coverage by mental healthcare of immigrant groups, especially refugees. This is reflected by lower treatment rates with any targeted medication and specifically mood stabilizers in all groups with a migration background compared to Swedish‐born patients, as well as by the highest risk of unfavorable antidepressant monotherapy in refugees. Furthermore, the preference for sedating drugs such as mirtazapine, olanzapine, and risperidone in refugees compared to Swedish‐born patients, while disregarding better‐tolerated first‐line options such as aripiprazole, requires attention. On a positive note, among more recently diagnosed patients initiating treatment in 2014–2018, comparable use of quetiapine across host and immigrant populations is assuring that gaps in treatment practices can be bridged in reasonable time periods. Finally, neglect of lithium was widespread in all population groups but especially so in young refugees with BD and must be addressed by public health measures in a timely manner.

## 6 Significant Outcomes

1. From incident diagnosis to 5 years later, immigrant groups and, particularly, refugees were at higher risk of not receiving any pharmacological treatment targeting bipolar disorder.
2. The use of mood stabilizers was considerably higher in Swedish‐born patients than in immigrant groups and especially refugees, while sedating drugs such as mirtazapine and olanzapine were more common among refugees.
3. Antidepressant monotherapy was common across all population groups but particularly overrepresented among refugees (20.3%).

## 7 Limitations

1. Clinical characteristics such as symptom presentation and severity could not be considered as data are based on Swedish national registries.
2. Immigrants are less likely to get in contact with the healthcare system, and rates of bipolar disorder identified by insurance data registers may therefore be too low.
3. Medication use was calculated on drug dispensing in Swedish pharmacies but did not account for treatment received during hospital stays.

## Author Contributions

A.K., H.T., and E.M.R. designed the study. E.M.R. obtained funding. A.K., A.T., and E.P. analyzed the data. A.K., R.A., A.A., and K.G. drafted the report. All authors interpreted the data, participated in the critical revision, and approved the final article.

## Ethics Statement

The project was approved by the Regional Ethical Review Board, Karolinska Institutet, Stockholm, Sweden (Review Number Dnr 2007/762–31 and Dnr 2021–06441‐02)).

## Conflicts of Interest

H.T. has received grants from Janssen and lecture fees from Gedeon Richter, Janssen, Lundbeck, and Otsuka, outside of the submitted work. Other authors declare that they have no potential conflicts of interest to disclose.

## Supporting information


Data S1.

## Data Availability

The data used in this study cannot be made publicly available due to privacy regulations. According to the General Data Protection Regulation, the Swedish law SFS 2018:218, the Swedish Data Protection Act, the Swedish Ethical Review Act, and the Public Access to Information and Secrecy Act, these types of sensitive data can only be made available for specific purposes, including research that meets the criteria for access to this sort of sensitive and confidential data as determined by a legal review. Readers may contact Professor Kristina Alexanderson (kristina.alexanderson@ki.se) regarding the data.
