# Supplementary material for: Treatment Patterns for Incident Bipolar Disorder Among Nonrefugee Immigrants, Refugees, Second‐Generation Immigrants, and Host Population in Sweden
Source: Bipolar Disord. 2025 Mar 7;27(3):192–204. doi: 10.1111/bdi.70007 (PMC12169100; doi:10.1111/bdi.70007)
Supplement: Supplementary file 1 — Data S1. [file BDI-27-192-s001.docx]

| **Register** | **Type of Information** | **Reference** |
| --- | --- | --- |
| Longitudinal Database  for Integration Studies (STATIV) | refugee status and migration background for definition of 1^st^-generation immigrant and refugee groups | (Statistics Sweden, 2018) |
| Swedish Multi-generation Register | parental countries of birth for definition of the Swedish-born and 2^nd^-generation immigrant groups | (Ekbom, 2011) |
| National Patient Register (NPR) | dates of visits and main- and secondary-diagnoses from secondary healthcare in Sweden (specialized outpatient visits and inpatient stays) | (Ludvigsson *et al.*, 2011) |
| Prescribed Drug Register (PDR) | substances with dosages und quantities dispensed in Swedish pharmacies by Anatomical Therapeutic Chemical (ATC) codes | (Wettermark *et al.*, 2007) |
| Longitudinal Integration Database for Health Insurance and Labor Market Studies (LISA) | sociodemographic variables such as sex, education, living situation, place of residence, country of birth | (Ludvigsson *et al.*, 2019) |
| Total Population Register | emigration from Sweden, assessed annually at the end of each year | (Ludvigsson *et al.*, 2016) |
| Micro-Data for Analyses of Social Insurance (MIDAS) | dates and diagnoses regarding sickness absence and disability pension | (Försäkringskassan, 2018) |
| Cause of Death Register | date of death | - (Brooke *et al.*, 2017) |

**Supplementary Table 1.** Swedish national registries linked to obtain relevant pseudonymized population-wide data. The unique personal identification number provided to every person living in Sweden was used for linkage.

**Supplementary Section**

|  | **First-Generation Immigrants** | | | |
| --- | --- | --- | --- | --- |
| **Characteristic** | **Non-refugee** | | **Refugees** | |
|  | n | % | n | % |
| **Country of Birth** | | | | |
| Iran | 108 | 5.4 | 183 | 15.5 |
| Bosnia | 13 | 0.6 | 147 | 12.4 |
| Iraq | 80 | 4.0 | 118 | 10.0 |
| Former Yugoslavia | 81 | 4.0 | 100 | 8.5 |
| Chile | 126 | 6.3 | 63 | 5.3 |
| Turkey | 67 | 3.3 | 34 | 2.9 |
| Poland | 230 | 11.4 | 33 | 2.8 |
| Russia | 38 | 1.9 | 32 | 2.7 |
| Somalia | 24 | 1.2 | 32 | 2.7 |
| Libanon | 32 | 1.6 | 23 | 1.9 |
| United States of America | 93 | 4.6 | 21 | 1.8 |
| United Kingdom | 100 | 5.0 | 13 | 1.1 |
| Germany | 77 | 3.8 |  |  |
| Colombia | 69 | 3.4 |  |  |
| India | 80 | 4.0 |  |  |
| **Duration of Residence** |  |  |  |  |
| 3 – 10 years | 537 | 26.7 | 318 | 26.9 |
| 11 – 20 years | 473 | 23.5 | 556 | 47.0 |
| > 20 years | 1003 | 49.8 | 308 | 26.1 |

**Supplementary Table 2.** Country of birth and duration of residence in Sweden of first‑generation immigrant groups (refugees and non-refugee immigrants). The 10 most common countries of birth respectively of the refugee and non-refugee immigrants are listed, ordered in descending numbers for the refugee group. Data are only shown for countries of birth with more than 10 individuals

| **Antidepressants** | **N06A** |
| --- | --- |
| SSRI | N06AB |
| SNRI | N06AX16, N06AX17, N06AX21 |
| Mirtazapine | N06AX11 |
| **Mood-Stabilizers** | **N05AN01, N03AG01, N03AX09, N03AF01, N03AF02** |
| Lithium | N05AN01 |
| Valproate | N03AG01 |
| Lamotrigine | N03AX09 |
| Carbamazepine, Oxcarbazepine | N03AF01, N03AF02 |
| **Oral Antipsychotics** | **N05A**, excluding Lithium |
| Quetiapine | N05AH04 |
| Olanzapine | N05AH03 |
| Aripiprazole | N05AX12 |
| Risperidone, Paliperidone | N05AX08, N05AX13 |
| **Long-acting Injectables** | |
| Aripiprazole, Olanzapine, Risperidone, Paliperidone, Haloperidol, Zuclopenthixol, Flupentixol, Perphenazine | N05AX12, N05AH03,  N05AX08, N05AX13,  N05AD01, N05AF05,  N05AB03, N05AF01 |
| **Sedatives** | **N05BA, N05CD, N05CF, N05CM06** |
| Benzodiazepines | N05BA, N05CD |
| Z-drugs | N05CF |
| Propiomazine | N05CM06 |

Abbreviations: SSRI = selective serotonin-reuptake inhibitors, SNRI = serotonin-noradrenaline-reuptake inhibitors

**Supplementary Table 3.** Drug classes and respective subclasses or substances analyzed, listed by ATC codes.

| **Antidepressants** | | | |  | **Antipsychotics** | | | | |
| --- | --- | --- | --- | --- | --- | --- | --- | --- | --- |
| **Name** | **ATC** | **n** | **%** |  | **Name** | **ATC** | **n** | **%** | |
| Bupropion | N06AX12 | 1031 | 3.62% |  | Levomepromazine | N05AA02 | 430 | | 1.51% |
| Amitriptyline | N06AA09 | 385 | 1.35% |  | Haloperidol | N05AD01 | 150 | | 0.53% |
| Clomipramine | N06AA04 | 316 | 1.11% |  | Flupentixol | N05AF01 | 80 | | 0.28% |
| Mianserine | N06AX03 | 264 | 0.93% |  | Zuclopentixol | N05AF05 | 76 | | 0.27% |
| Agomelatine | N06AX22 | 246 | 0.86% |  | Chlorprotixen | N05AF03 | 70 | | 0.25% |
| Vortioxetine | N06AX26 | 135 | 0.47% |  | Ziprasidon | N05AE04 | 45 | | 0.16% |
| Reboxetine | N06AX18 | 97 | 0.34% |  | Perfenazine | N05AB03 | 37 | | 0.13% |
| Moclobemide | N06AG02 | 40 | 0.14% |  | Melperone | N05AD03 | 11 | | 0.04% |
| Nortriptyline | N06AA10 | 22 | 0.08% |  | Lurasidone | N05AE05 | 8 | | 0.03% |
| Tranylcypromine | N06AF04 | 7 | 0.02% |  | Dixyrazine | N05AB01 | 6 | | 0.02% |

**Supplementary Table 4.** List of the ten most common not-analyzed drugs respectively among antipsychotics and antidepressants used by subjects with incident bipolar disorder 3 months after diagnosis.

| **Time-Point** | | **Swedish-born** | **Second-Generation**  **Immigrants** | **First-Generation Immigrants** | |
| --- | --- | --- | --- | --- | --- |
|  |  |  |  | **Non-refugee** | **Refugees** |
| **Lack of Treatment and Drug Classes** | |  |  |  |  |
| No Treatment | 0.25 | 17.25 (16.81-17.70) | 21.06 (19.70-22.50) | 23.09 (21.33-24.99) | 26.75 (24.35-29.39) |
|  | 1 | 25.90 (25.38-26.43) | 30.98 (29.43-32.61) | 35.00 (32.95-37.17) | 38.91 (36.19-41.83) |
|  | 3 | 35.11 (34.50-35.74) | 40.19 (38.41-42.06) | 45.17 (42.85-47.63) | 49.54 (46.50-52.78) |
| Antidepressants | 0.25 | 51.59 (51.00-52.19) | 46.38 (44.63-48.19) | 43.25 (41.25-45.35) | 42.41 (39.88-45.11) |
|  | 1 | 45.24 (44.65-45.84) | 39.37 (37.62-41.20) | 36.89 (34.91-38.98) | 37.26 (34.70-40.00) |
|  | 3 | 40.06 (39.44-40.68) | 32.98 (31.16-34.91) | 32.15 (30.08-34.37) | 31.07 (28.45-33.94) |
| Mood-Stabilizers | 0.25 | 51.25 (50.64-51.85) | 47.88 (46.08-49.75) | 44.53 (42.42-46.74) | 35.36 (32.75-38.18) |
|  | 1 | 45.94 (45.34-46.55) | 42.06 (40.27-43.92) | 38.18 (36.11-40.38) | 29.82 (27.31-32.55) |
|  | 3 | 37.05 (36.43-37.67) | 34.45 (32.62-36.39) | 30.12 (28.05-32.34) | 22.60 (20.19-25.30) |
| Oral Antipsychotics | 0.25 | 24.44 (23.92-24.96) | 26.44 (24.87-28.12) | 25.90 (24.04-27.90) | 27.49 (25.01-30.21) |
|  | 1 | 20.04 (19.56-20.54) | 20.32 (18.87-21.88) | 19.15 (17.47-20.98) | 19.51 (17.31-21.99) |
|  | 3 | 17.81 (17.31-18.32) | 17.48 (16.02-19.08) | 17.80 (16.04-19.75) | 16.43 (14.26-18.93) |
| Sedatives | 0.25 | 29.29 (28.76-29.83) | 28.94 (27.37-30.60) | 28.37 (26.57-30.29) | 26.67 (24.39-29.17) |
|  | 1 | 25.86 (25.35-26.38) | 25.35 (23.82-26.97) | 22.45 (20.80-24.24) | 21.07 (18.98-23.40) |
|  | 3 | 24.11 (23.58-24.65) | 23.61 (21.97-25.38) | 21.78 (20.00-23.72) | 20.37 (18.12-22.90) |
| **Antidepressants - Subclasses** | | | | | |
| Antidepressant-  Monotherapy | 0.25 | 17.66 (17.21-18.12) | 16.82 (15.50-18.26) | 17.72 (16.17-19.42) | 20.32 (18.22-22.65) |
|  | 1 | 17.50 (17.04-17.96) | 15.77 (14.47-17.19) | 15.92 (14.42-17.59) | 19.17 (17.07-21.52) |
|  | 3 | 17.87 (17.38-18.37) | 14.67 (13.28-16.20) | 14.45 (12.87-16.22) | 17.82 (15.61-20.34) |
| SSRI | 0.25 | 33.28 (32.72-33.85) | 28.58 (26.99-30.27) | 28.09 (26.22-30.08) | 25.19 (22.88-27.74) |
|  | 1 | 27.16 (26.62-27.70) | 22.55 (21.04-24.17) | 22.82 (21.06-24.73) | 22.13 (19.89-24.62) |
|  | 3 | 22.41 (21.88-22.96) | 17.78 (16.31-19.39) | 17.83 (16.09-19.75) | 16.25 (14.13-18.68) |
| SNRI | 0.25 | 11.8 (11.4-12.2) | 10.8 ( 9.7-12.0) | 9.0 ( 7.9-10.2) | 8.7 ( 7.3-10.3) |
|  | 1 | 10.33 ( 9.98-10.70) | 8.86 ( 7.88- 9.96) | 7.40 ( 6.40- 8.57) | 7.69 ( 6.39- 9.25) |
|  | 3 | 8.85 ( 8.50- 9.21) | 6.86 ( 5.93- 7.94) | 6.45 ( 5.44- 7.65) | 7.14 ( 5.80- 8.78) |
| Mirtazapine | 0.25 | 6.46 (6.17-6.76) | 6.49 (5.66-7.43) | 6.27 (5.36-7.35) | 7.89 (6.61-9.43) |
|  | 1 | 4.71 (4.47-4.97) | 4.44 (3.75-5.25) | 4.53 (3.75-5.48) | 6.44 (5.25-7.90) |
|  | 3 | 3.98 (3.74-4.22) | 3.59 (2.92-4.40) | 3.66 (2.90-4.63) | 4.76 (3.66-6.19) |
| **Oral Antipsychotics - Subclasses** | | | | | |
| Quetiapine | 0.25 | 14.14 (13.73-14.57) | 13.96 (12.75-15.27) | 12.98 (11.58-14.55) | 12.87 (11.06-14.97) |
|  | 1 | 12.48 (12.08-12.89) | 11.67 (10.54-12.92) | 11.26 ( 9.93-12.77) | 9.74 ( 8.15-11.64) |
|  | 3 | 10.95 (10.54-11.37) | 10.49 ( 9.32-11.79) | 10.46 ( 9.08-12.05) | 8.89 ( 7.28-10.86) |
| Olanzapine | 0.25 | 6.23 (5.95- 6.53) | 8.02 (7.08- 9.08) | 7.97 (6.89- 9.22) | 9.35 (7.85-11.15) |
|  | 1 | 3.76 (3.54- 3.99) | 4.23 (3.53- 5.07) | 4.04 (3.27- 5.00) | 5.51 (4.34- 6.99) |
|  | 3 | 3.04 (2.82- 3.27) | 3.12 (2.48- 3.92) | 3.51 (2.73- 4.53) | 4.56 (3.44- 6.04) |
| Aripiprazole | 0.25 | 2.22 (2.04-2.40) | 2.25 (1.78-2.85) | 2.53 (1.93-3.32) | 1.81 (1.19-2.76) |
|  | 1 | 2.43 (2.25-2.63) | 2.83 (2.28-3.50) | 2.40 (1.80-3.19) | 2.19 (1.48-3.24) |
|  | 3 | 2.62 (2.41-2.84) | 2.48 (1.93-3.19) | 2.26 (1.63-3.12) | 1.66 (1.03-2.69) |
| Risperidone/  Paliperidone | 0.25 | 1.09 (0.97-1.22) | 1.28 (0.94-1.75) | 1.39 (0.99-1.97) | 1.84 (1.21-2.80) |
|  | 1 | 0.72 (0.63-0.83) | 0.90 (0.61-1.33) | 0.87 (0.56-1.34) | 1.02 (0.60-1.74) |
|  | 3 | 0.56 (0.47-0.67) | 0.55 (0.32-0.94) | 1.13 (0.75-1.71) | 1.08 (0.60-1.93) |
| **Time-Point** | | **Swedish-born** | **Second-Generation**  **Immigrants** | **First-Generation Immigrants** | |
|  |  |  |  | **Non-refugee** | **Refugees** |
| **Mood-Stabilizers - Subclasses** | | | |  |  |
| Lithium | 0.25 | 13.17 (12.77-13.59) | 12.99 (11.81-14.29) | 13.06 (11.71-14.57) | 11.24 ( 9.61-13.15) |
|  | 1 | 16.58 (16.13-17.04) | 16.64 (15.30-18.09) | 15.89 (14.40-17.54) | 13.63 (11.83-15.71) |
|  | 3 | 14.61 (14.16-15.07) | 14.29 (12.96-15.75) | 12.02 (10.63-13.60) | 11.29 ( 9.53-13.36) |
| Valproate | 0.25 | 6.97 (6.66-7.28) | 7.73 (6.80-8.79) | 7.57 (6.50-8.81) | 5.90 (4.71-7.41) |
|  | 1 | 4.97 (4.71-5.24) | 4.81 (4.06-5.70) | 4.23 (3.44-5.21) | 3.53 (2.61-4.78) |
|  | 3 | 3.78 (3.54-4.04) | 4.35 (3.59-5.29) | 4.07 (3.23-5.14) | 3.17 (2.24-4.48) |
| Lamotrigine | 0.25 | 32.57 (32.00-33.14) | 29.07 (27.49-30.73) | 26.29 (24.45-28.26) | 20.17 (18.02-22.57) |
|  | 1 | 27.69 (27.15-28.24) | 23.98 (22.48-25.57) | 20.91 (19.20-22.78) | 15.03 (13.12-17.23) |
|  | 3 | 22.03 (21.50-22.57) | 18.92 (17.46-20.51) | 16.94 (15.27-18.79) | 10.59 ( 8.86-12.66) |
| Carbamazepine/  Oxcarbazepine | 0.25 | 0.61 (0.52-0.71) | 0.46 (0.27-0.78) | 0.35 (0.17-0.71) | 0.40 (0.17-0.97) |
|  | 1 | 0.39 (0.32-0.47) | 0.57 (0.36-0.91) | 0.37 (0.18-0.76) | 0.41 (0.17-1.02) |
|  | 3 | 0.42 (0.34-0.52) | 0.58 (0.34-0.97) | 0.42 (0.19-0.90) | 0.47 (0.18-1.21) |
| **Sedatives - Subclasses** | | | | | |
| Benzodiazepine | 0.25 | 11.86 (11.49-12.24) | 12.45 (11.34-13.66) | 9.00 ( 7.92-10.23) | 8.96 ( 7.58-10.58) |
|  | 1 | 10.15 ( 9.81-10.51) | 10.65 ( 9.60-11.82) | 7.54 ( 6.53- 8.71) | 7.18 ( 5.92- 8.71) |
|  | 3 | 9.59 ( 9.23- 9.96) | 9.85 ( 8.74-11.10) | 7.37 ( 6.29- 8.63) | 6.46 ( 5.16- 8.09) |
| Z-Drugs | 0.25 | 17.99 (17.55-18.45) | 18.15 (16.81-19.59) | 16.85 (15.37-18.46) | 16.64 (14.75-18.77) |
|  | 1 | 16.57 (16.14-17.01) | 16.62 (15.31-18.05) | 14.81 (13.41-16.36) | 13.94 (12.19-15.93) |
|  | 3 | 16.00 (15.54-16.47) | 15.30 (13.93-16.81) | 14.93 (13.40-16.63) | 13.15 (11.30-15.31) |
| Propiomazine | 0.25 | 10.14 (9.79-10.51) | 9.85 (8.81-11.00) | 10.60 (9.37-12.00) | 9.42 (7.93-11.20) |
|  | 1 | 8.26 (7.94- 8.59) | 7.52 (6.59- 8.58) | 7.53 (6.48- 8.75) | 7.12 (5.80- 8.75) |
|  | 3 | 7.23 (6.91- 7.57) | 6.33 (5.39- 7.43) | 6.18 (5.16- 7.41) | 7.71 (6.22- 9.55) |
| **Mood Stabilizers and/or Antipsychotcis** | | | |  |  |
|  | 0.25 | 62.45 (61.87-63.03) | 59.60 (57.83-61.43) | 56.07 (53.97-58.25) | 49.92 (47.17-52.84) |
|  | 1 | 50.79 (50.20-51.39) | 47.72 (45.94-49.57) | 43.29 (41.21-45.48) | 36.65 (34.04-39.46) |
|  | 3 | 33.80 (33.25-34.37) | 30.35 (28.73-32.07) | 28.45 (26.57-30.47) | 21.87 (19.66-24.33) |

**Supplementary Table 5.** Marginal means with corresponding 95% confidence intervals estimated by generalized estimating equations adjusted for socio-demographic and comorbidity covariates. Abbreviations: SSRI = selective serotonin-reuptake inhibitors, SNRI = serotonin-noradrenaline-reuptake inhibitors.

|  |  |  |  |
| --- | --- | --- | --- |
|  |  |  |  |

|  | **Antidepressants** | | **SSRI** | | **SNRI** | | **Mirtazapine** | | **Antidepressant**  **Monotherapy** | | |
| --- | --- | --- | --- | --- | --- | --- | --- | --- | --- | --- | --- |
| **Effect** | F | p | F | p | F | p | F | p | F | p | |
| Time-Point | 39.4 | <0.0001 | 38.0 | <0.0001 | 8.3 | <0.0001 | 14.4 | <0.0001 | 2.0 | 0.0266 | |
| Group | 52.7 | <0.0001 | 29.3 | <0.0001 | 14.0 | 0.0005 | 2.6 | 0.0492 | 6.7 | 0.0002 | |
| Age | 120.4 | <0.0001 | 9.6 | 0.0019 | 117.8 | <0.0001 | 158.7 | <0.0001 | 128.3 | <0.0001 | |
| Time-Point * Group | 0.9 | n.s. | 0.7 | n.s. | 0.7 | n.s. | 0.8 | n.s. | 1.2 | n.s. | |
| Time-Point * Age | 0.7 | n.s. |  |  |  |  |  |  |  |  | |
| Group * Age | 4.6 | 0.003 |  |  |  |  |  |  |  |  | |
| Time-Point * Goup * Age | 0.6 | n.s. |  |  |  |  |  |  |  |  | |
|  | **Mood Stabilizers** | | **Lamotrigine** | | **Lithium** | | **Valproate** | | **Carbamazepine** | | |
| Time-Point | 53.4 | <0.0001 | 34.8 | <0.0001 | 14.2 | <0.0001 | 14.8 | <0.0001 | 0.6 | n.s. | |
| Group | 60.2 | <0.0001 | 57.1 | <0.0001 | 5.4 | 0.001 | 1.8 | 0.141 | 1.4 | n.s. | |
| Age | 16.8 | <0.0001 | 78.3 | <0.0001 | 0.1 | 0.738 | 4.6 | 0.033 | 1.9 | n.s. | |
| Time-Point * Group | 1.0 | n.s. | 1.1 | n.s. | 1.0 | 0.497 | 1.4 | 0.052 | 1.3 | n.s. | |
| Time-Point * Age |  |  |  |  | 1.1 | n.s. | 2.7 | 0.002 |  | | |
| Group * Age |  |  |  |  | 0.9 | n.s. | 1.0 | n.s. |  | | |
| Time-Point * Goup * Age |  |  |  |  | 1.8 | 0.004 | 1.8 | 0.005 |  | | |
|  | **Antipsychotics** | | **Quetiapine** | | **Olanzapine** | | **Risperidone** | | **Aripiprazole** | | |
| Time-Point | 32.2 | <0.0001 | 7.1 | <0.0001 | 32.0 | <0.0001 | 3.3 | 0.0002 | 0.7 | | n.s. |
| Group | 0.1 | n.s. | 3.2 | 0.021 | 5.4 | 0.001 | 4.4 | 0.0039 | 1.7 | | n.s. |
| Age | 13.3 | 0.0003 | 0.5 | n.s. | 4.9 | 0.027 | 17.5 | <0.0001 | 8.9 | | 0.0028 |
| Time-Point * Group | 1.2 | n.s. | 0.9 | n.s. | 0.8 | n.s. | 1.1 | n.s. | 0.8 | | n.s. |
| Time-Point * Age |  |  |  |  | 1.4 | n.s. |  |  |  | | |
| Group * Age |  |  |  |  | 3.3 | 0.021 |  |  |  | | |
| Time-Point * Goup * Age |  |  |  |  | 0.9 | n.s. |  |  |  | | |
|  | **No Treatment** | | **Sedatives** | | **Benzodiazepines** | | **Z-Drugs** | | **Propiomazine** | | |
| Time-Point | 151.9 | <0.0001 | 16.7 | <0.0001 | 5.3 | <0.0001 | 4.8 | <0.0001 | 11.4 | <0.0001 | |
| Group | 104.9 | <0.0001 | 6.1 | 0.0004 | 13.5 | <0.0001 | 2.8 | 0.0361 | 1.8 | n.s. | |
| Age | 101.1 | <0.0001 | 534.1 | <0.0001 | 236.1 | <0.0001 | 386.0 | <0.0001 | 145.0 | <0.0001 | |
| Time-Point * Group | 0.6 | n.s. | 1.1 | n.s. | 1.1 | n.s. | 0.7 | n.s. | 0.7 | n.s. | |
|  | **Mood Stabilizers and/or Antipsychotics** | | | | | | | | | | |
| Time-Point | 240.3 | <0.001 |  |  |  | | | | | | |
| Group | 54.0 | <0.001 |  |  |  |  |  |  |  |  |  |
| Time-Point * Group | 1.5 | 0.038 |  |  |  |  |  |  |  |  |  |

**Supplementary Table 6.** Joint test results of generalized estimation equations, adjusted for socio-demographic and comorbidity covariates. Main and interaction effects for time-point, population-group based on history of immigration, and age-groups (16-35 vs 36‑65) are presented. Insignificant interactions with age-group were dropped from the models. Only significant p-values are listed. Abbreviations: SSRI = selective serotonin-reuptake inhibitors, SNRI = serotonin-noradrenaline-reuptake inhibitors, n.s. = not significant.

| **A)** | | **Time-Point** | | **Swedish-born** | | | **Second-Generation**  **Immigrants** | | | **First-Generation Immigrants** | | | | | | | |
| --- | --- | --- | --- | --- | --- | --- | --- | --- | --- | --- | --- | --- | --- | --- | --- | --- | --- |
|  |  |  |  |  |  |  |  |  |  | **Non-refugee** | | | | | **Refugees** | | |
| **Age 16-35** | | | | | | | | | | | | | | | | | |
| Antidepressants | 0.25 | | | 46.21 (45.41-47.02) | | | 40.41 (38.35-42.57) | | | | 36.59 (33.70-39.72) | | | 32.67 (29.13-36.63) | | | |
|  | 1 | | | 39.35 (38.57-40.16) | | | 33.12 (31.10-35.28) | | | | 29.85 (27.05-32.93) | | | 29.92 (26.36-33.97) | | | |
|  | 3 | | | 34.25 (33.42-35.10) | | | 27.06 (24.96-29.33) | | | | 25.17 (22.25-28.47) | | | 24.61 (20.99-28.84) | | | |
| Lithium | 0.25 | | | 12.02 (11.50-12.56) | | | 12.38 (11.00-13.94) | | | | 13.02 (11.04-15.36) | | | 13.23 (10.71-16.35) | | | |
|  | 1 | | | 15.46 (14.88-16.07) | | | 15.37 (13.83-17.09) | | | | 15.81 (13.62-18.35) | | | 13.21 (10.63-16.41) | | | |
|  | 3 | | | 13.61 (13.01-14.23) | | | 12.79 (11.25-14.55) | | | | 11.66 ( 9.63-14.12) | | | 10.64 ( 8.15-13.90) | | | |
| Valproate | 0.25 | | | 6.36 (5.97-6.78) | | | 6.21 (5.23-7.37) | | | | 7.31 (5.84-9.16) | | | 7.36 (5.52-9.81) | | | |
|  | 1 | | | 4.21 (3.89-4.56) | | | 4.08 (3.27-5.08) | | | | 3.65 (2.62-5.09) | | | 3.50 (2.26-5.43) | | | |
|  | 3 | | | 2.76 (2.48-3.08) | | | 3.74 (2.90-4.83) | | | | 3.90 (2.72-5.59) | | | 2.92 (1.72-4.97) | | | |
| Oral Olanzapine | 0.25 | | | 5.03 (4.69- 5.40) | | | 7.34 (6.29- 8.57) | | | | 7.76 (6.23- 9.67) | | | 9.22 (7.14-11.90) | | | |
|  | 1 | | | 2.86 (2.60- 3.15) | | | 4.00 (3.21- 4.99) | | | | 4.51 (3.36- 6.05) | | | 5.44 (3.85- 7.69) | | | |
|  | 3 | | | 2.31 (2.06- 2.60) | | | 2.47 (1.81- 3.38) | | | | 3.14 (2.06- 4.79) | | | 3.33 (2.06- 5.38) | | | |
| **Age 36-65** | | | | | | | | | | | | | | | | | |
| Antidepressants | 0.25 | | | 58.62 (57.75-59.51) | | | 54.50 (51.56-57.61) | | | | 51.03 (48.20-54.02) | | | 52.86 (49.22-56.77) | | | |
|  | 1 | | | 52.85 (51.96-53.75) | | | 48.68 (45.67-51.89) | | | | 44.69 (41.84-47.73) | | | 45.35 (41.66-49.36) | | | |
|  | 3 | | | 47.53 (46.61-48.48) | | | 42.08 (38.93-45.49) | | | | 39.62 (36.64-42.84) | | | 38.08 (34.27-42.30) | | | |
| Lithium | 0.25 | | | 14.50 (13.88-15.15) | | | 13.42 (11.44-15.74) | | | | 13.48 (11.64-15.60) | | | 9.90 ( 7.86-12.47) | | | |
|  | 1 | | | 17.86 (17.18-18.57) | | | 18.19 (15.89-20.81) | | | | 16.44 (14.41-18.75) | | | 14.37 (11.92-17.34) | | | |
|  | 3 | | | 15.77 (15.10-16.47) | | | 16.44 (14.17-19.08) | | | | 12.69 (10.80-14.90) | | | 12.11 ( 9.74-15.06) | | | |
| Valproate | 0.25 | | | 7.71 (7.24- 8.20) | | | 10.03 (8.28-12.15) | | | | 8.05 (6.57- 9.87) | | | 4.69 (3.27- 6.73) | | | |
|  | 1 | | | 5.90 (5.49- 6.35) | | | 5.77 (4.43- 7.51) | | | | 4.90 (3.76- 6.40) | | | 3.60 (2.37- 5.47) | | | |
|  | 3 | | | 5.03 (4.63- 5.47) | | | 5.13 (3.81- 6.91) | | | | 4.40 (3.25- 5.95) | | | 3.46 (2.18- 5.51) | | | |
| Oral Olanzapine | 0.25 | | | 7.75 (7.29- 8.25) | | | 8.20 (6.65-10.12) | | | | 8.63 (7.10-10.50) | | | 9.84 (7.76-12.48) | | | |
|  | 1 | | | 4.88 (4.50- 5.29) | | | 4.11 (3.01- 5.60) | | | | 3.92 (2.91- 5.29) | | | 5.75 (4.14- 7.97) | | | |
|  | 3 | | | 3.94 (3.58- 4.33) | | | 4.01 (2.87- 5.60) | | | | 4.11 (3.00- 5.63) | | | 5.66 (3.97- 8.05) | | | |
| **B) Stratification by Age-Group** | | | | | **Antidepressants** | | | **Lithium** | | | | **Valproate** | | | | **Olanzapine** | |
| Time-Point | | | 16 – 35 | | 17.5 | <0.0001 | | 6.4 | <0.0001 | | | 11.3 | <0.0001 | | | 13.7 | <0.0001 |
| Group | | |  |  | 36.2 | <0.0001 | | 1 | n.s. | | | 0.8 | n.s. | | | 0.9 | n.s. |
| Time-Point * Group | | |  |  | 0.6 | n.s. | | 1.3 | n.s. | | | 1.8 | 0.0029 | | | 0.7 | n.s. |
| Time-Point | | | 36 – 65 | | 25.8 | <0.0001 | | 9.1 | <0.0001 | | | 5.5 | <0.0001 | | | 19 | <0.0001 |
| Group | | |  |  | 20.2 | <0.0001 | | 5.6 | 0.0008 | | | 2.4 | n.s. | | | 6.7 | 0.0002 |
| Time-Point * Group | | |  |  | 1.0 | n.s. | | 1.4 | 0.08 | | | 1.4 | n.s. | | | 0.9 | n.s. |

**Supplementary Table 7.** Results of generalized estimation equations, adjusted for socio-demographic and comorbidity covariates, respectively for patient cohorts grouped by age (16‑35 and 36-65) at incident diagnosis of bipolar disorder. Models were estimated for outcomes with significant interactions between population-groups based on immigration history and age-groups. A) Marginal means with corresponding 95% confidence intervals, B) joint test results.

|  | No treatment | Antidepressants | Mood stabilizers | Antipsychotics |
| --- | --- | --- | --- | --- |
|  | RR, 95% CI | RR, 95% CI | RR, 95% CI | RR, 95% CI |
| Sociodemographic variables |  |  |  |  |
| Sex |  |  |  |  |
| Women vs Men | 1.09, 1.07-1.12 | 0.87, 0.85-0.89 | 0.99, 0.97-1.02 | 1.08, 1.05-1.12 |
|  |  |  |  |  |
| Age |  |  |  |  |
| 16-35 vs 36-65 | 0.86, 0.84-0.89 | 1.18, 1.16-1.21 | 0.95, 0.93-0.98 | 1.07, 1.03-1.11 |
|  |  |  |  |  |
| Education years |  |  |  |  |
| Missing vs 0-9 years | 1.22, 1.12-1.32 | 1.07, 0.98-1.17 | 0.94, 0.86-1.03 | 0.82, 0.73-0.92 |
| Missing vs 10-12 years | 1.05, 0.96-1.14 | 1.21, 1.11-1.32 | 1.07, 0.98-1.16 | 0.83, 0.74-0.93 |
| Missing vs >12 years | 0.90, 0.83-0.98 | 1.29, 1.18-1.41 | 1.14, 1.05-1.24 | 0.84, 0.75-0.95 |
|  |  |  |  |  |
| Family Situation |  |  |  |  |
| Single vs Single,with children | 1.02, 0.98-1.07 | 1.01, 0.97-1.04 | 1.06, 1.02-1.09 | 0.94, 0.89-1.00 |
| Single vs Cohabitant | 0.78, 0.73-0.82 | 1.14, 1.10-1.18 | 1.12, 1.08-1.17 | 1.12, 1.06-1.19 |
| Single vs Cohabitant, with children | 0.83, 0.80-0.86 | 1.12, 1.10-1.15 | 1.16, 1.13-1.19 | 0.99, 0.95-1.03 |
|  |  |  |  |  |
| Sickness absence |  |  |  |  |
| No vs 1-90 | 0.83, 0.79-0.87 | 1.20, 1.17-1.23 | 1.09, 1.06-1.13 | 1.16, 1.11-1.23 |
| No vs 91-365 | 0.89, 0.87-0.92 | 1.19, 1.16-1.21 | 1.02, 0.99-1.04 | 1.06, 1.02-1.10 |
|  |  |  |  |  |
| Disability pension |  |  |  |  |
| No vs Yes | 0.93, 0.89-0.97 | 1.20, 1.17-1.24 | 0.85, 0.82-0.88 | 0.95, 0.90-1.01 |
| Comorbidities ( No vs Yes) |  |  |  |  |
| Substance use | 1.10, 1.06-1.14 | 0.91, 0.89-0.94 | 0.92, 0.89-0.95 | 1.13, 1.08-1.19 |
| Depression | 0.80, 0.78-0.82 | 1.28, 1.26-1.31 | 1.11, 1.09-1.13 | 1.01, 0.97-1.04 |
| Anxiety disorder | 0.87, 0.84-0.89 | 1.16, 1.14-1.18 | 0.98, 0.96-1.00 | 1.16, 1.12-1.20 |
| PTSD | 1.18, 1.10-1.27 | 0.93, 0.87-0.99 | 0.87, 0.80-0.94 | 0.96, 0.86-1.07 |
| Nervous system disorder | 0.95, 0.91-1.00 | 1.02, 0.99-1.05 | 1.01, 0.97-1.04 | 1.04, 0.98-1.10 |
| Cancer | 1.02, 0.97-1.07 | 1.01, 0.97-1.04 | 0.94, 0.90-0.98 | 0.95, 0.89-1.02 |
| Circulatory system disorder | 1.04, 0.99-1.10 | 1.02, 0.98-1.05 | 0.93, 0.89-0.98 | 0.99, 0.93-1.06 |
| Musculoskeletal disorder | 0.99, 0.96-1.03 | 1.00, 0.98-1.03 | 0.98, 0.96-1.01 | 1.06, 1.02-1.11 |
| Obesity/diabetes | 0.80, 0.74-0.87 | 1.15, 1.11-1.20 | 1.06, 1.00-1.12 | 1.14, 1.04-1.24 |
| History of suicide attempts | 0.98, 0.94-1.03 | 1.04, 1.00-1.07 | 1.04, 1.00-1.09 | 1.14, 1.07-1.21 |

**Supplementary Table 8.** Results of generalized estimation equations, effects of socio-demographic and comorbidity covariates on medication use. Relative risk (RR) with 95% confidence intervals (CI) are presented across all population-groups, respectively adjusted for all other covariates.

| **A)** | | **Time-Point** | | **2006 - 2009** | | | | | | **2010 - 2013** | | | | | | **2014 - 2018** | | |
| --- | --- | --- | --- | --- | --- | --- | --- | --- | --- | --- | --- | --- | --- | --- | --- | --- | --- | --- |
| Antidepressants | | 0.25 | | 53.07 (52.06-54.09) | | | | | | 50.35 (49.47-51.25) | | | | | | 48.14 (47.32-48.99) | | |
|  |  | 1 | | 45.84 (44.84-46.86) | | | | | | 43.90 (43.03-44.80) | | | | | | 42.45 (41.61-43.32) | | |
|  |  | 3 | | 39.01 (38.04-40.02) | | | | | | 39.34 (38.48-40.23) | | | | | | 37.53 (36.50-38.60) | | |
| Mood-Stabilizers | | 0.25 | | 46.11 (45.05-47.18) | | | | | | 49.28 (48.36-50.22) | | | | | | 52.78 (51.93-53.65) | | |
|  |  | 1 | | 41.65 (40.62-42.70) | | | | | | 43.22 (42.32-44.14) | | | | | | 47.39 (46.51-48.28) | | |
|  |  | 3 | | 33.92 (32.93-34.94) | | | | | | 35.01 (34.14-35.90) | | | | | | 37.78 (36.76-38.83) | | |
| Antipsychotics | | 0.25 | | 21.14 (20.28-22.04) | | | | | | 25.28 (24.48-26.10) | | | | | | 26.90 (26.14-27.68) | | |
|  |  | 1 | | 16.89 (16.10-17.71) | | | | | | 20.13 (19.39-20.88) | | | | | | 22.08 (21.34-22.84) | | |
|  |  | 3 | | 15.80 (15.03-16.61) | | | | | | 18.87 (18.15-19.61) | | | | | | 17.69 (16.84-18.58) | | |
| Lithium | | 0.25 | | 14.61 (13.89-15.38) | | | | | | 12.05 (11.47-12.67) | | | | | | 12.93 (12.37-13.52) | | |
|  |  | 1 | | 17.61 (16.82-18.43) | | | | | | 15.25 (14.60-15.93) | | | | | | 16.69 (16.04-17.36) | | |
|  |  | 3 | | 14.57 (13.84-15.34) | | | | | | 13.76 (13.14-14.41) | | | | | | 14.63 (13.90-15.40) | | |
| **B)** | | **Antidepressants** | | | | **Mood-Stabilizers** | | | | **Lithium** | | | **Antipsychotics** | | | |  |  |
| **Effect** | | F | | p | | F | p | | | F | | p | F | p | | |  |  |
| Time-Point | | 161.1 | | | <0.0001 | 189.8 | | <0.0001 | | 53.4 | <0.0001 | | 69.2 | | | <0.0001 |  |  |
| Time-Period | | 4.8 | | | 0.0085 | 15.6 | | <0.0001 | | 6.5 | 0.0015 | | 33.4 | | | <0.0001 |  |  |
| Time-Point * Time-Period | | 1.6 | | | 0.0396 | 2.0 | | 0.0041 | | 1.6 | 0.0448 | | 2.7 | | | <0.0001 |  |  |

**Supplementary Table 9.** Results of generalized estimation equations, adjusted for socio-demographic and comorbidity covariates, respectively for patient cohorts grouped by time‑period of diagnosis, 2006-2009, 2010-2013 and 2014-2018. A) Marginal means with corresponding 95% confidence intervals, B) joint test results.

**
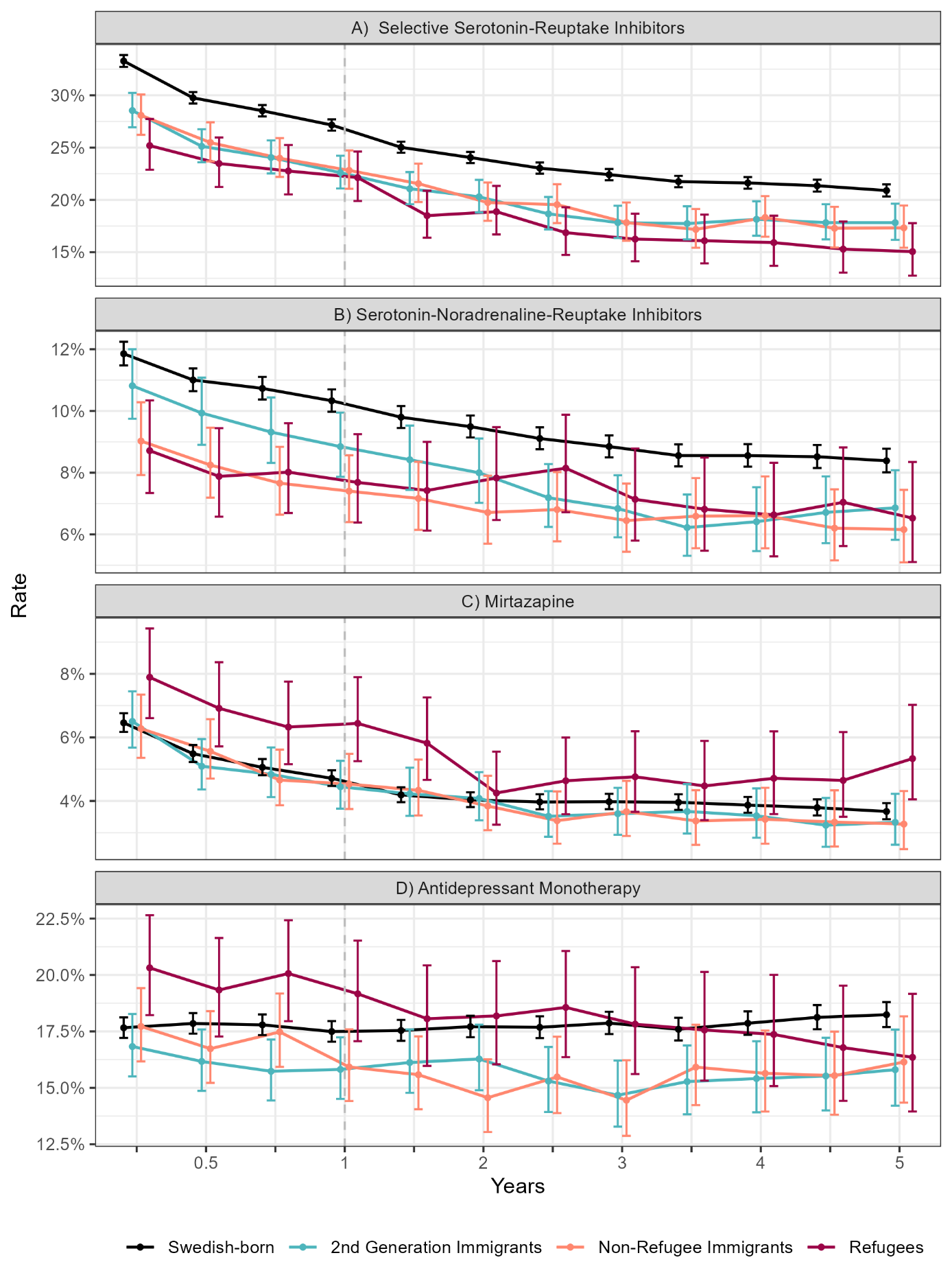
****Supplementary Figure 1.** Marginal means with corresponding 95% confidence intervals of estimated rates of antidepressant use stratified by drug subclasses, as well as monotherapy of any antidepressant. Rates are adjusted for socio-demographic and comorbidity covariates and are pictured over the observation time of 5 years following diagnosis with bipolar disorder. Colored lines correspond to groups based on history of immigration. The group of second-generation immigrants was born in Sweden, however, to parents with personal migration history.


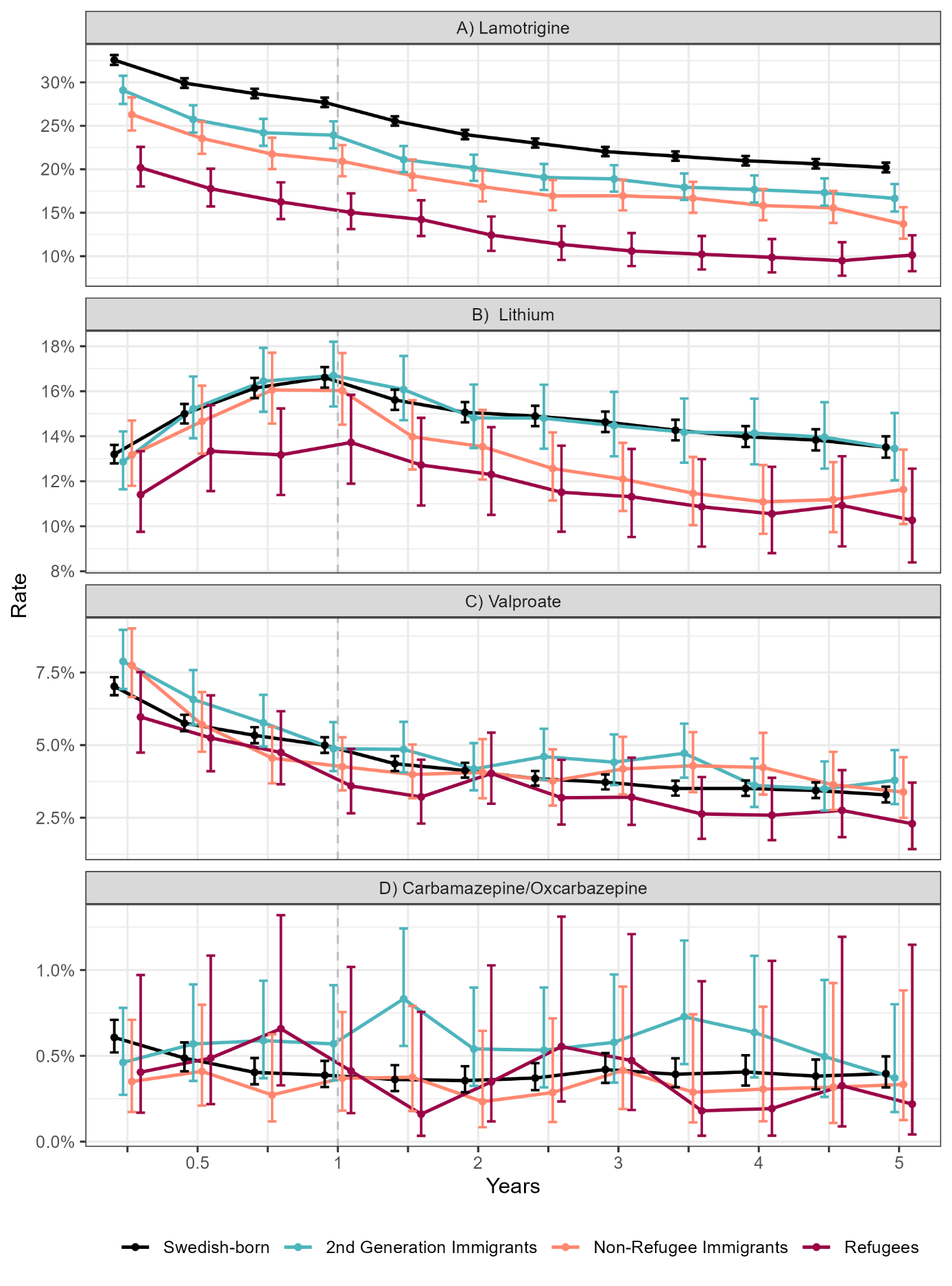


**Supplementary Figure 2.** Marginal means with corresponding 95% confidence intervals of estimated rates of mood-stabilizer use stratified by drug subclasses. Rates are adjusted for socio-demographic and comorbidity covariates and are pictured over the observation time of 5 years following diagnosis with bipolar disorder. Colored lines correspond to history of immigration. The group of second-generation immigrants was born in Sweden, however, to parents with personal migration history.


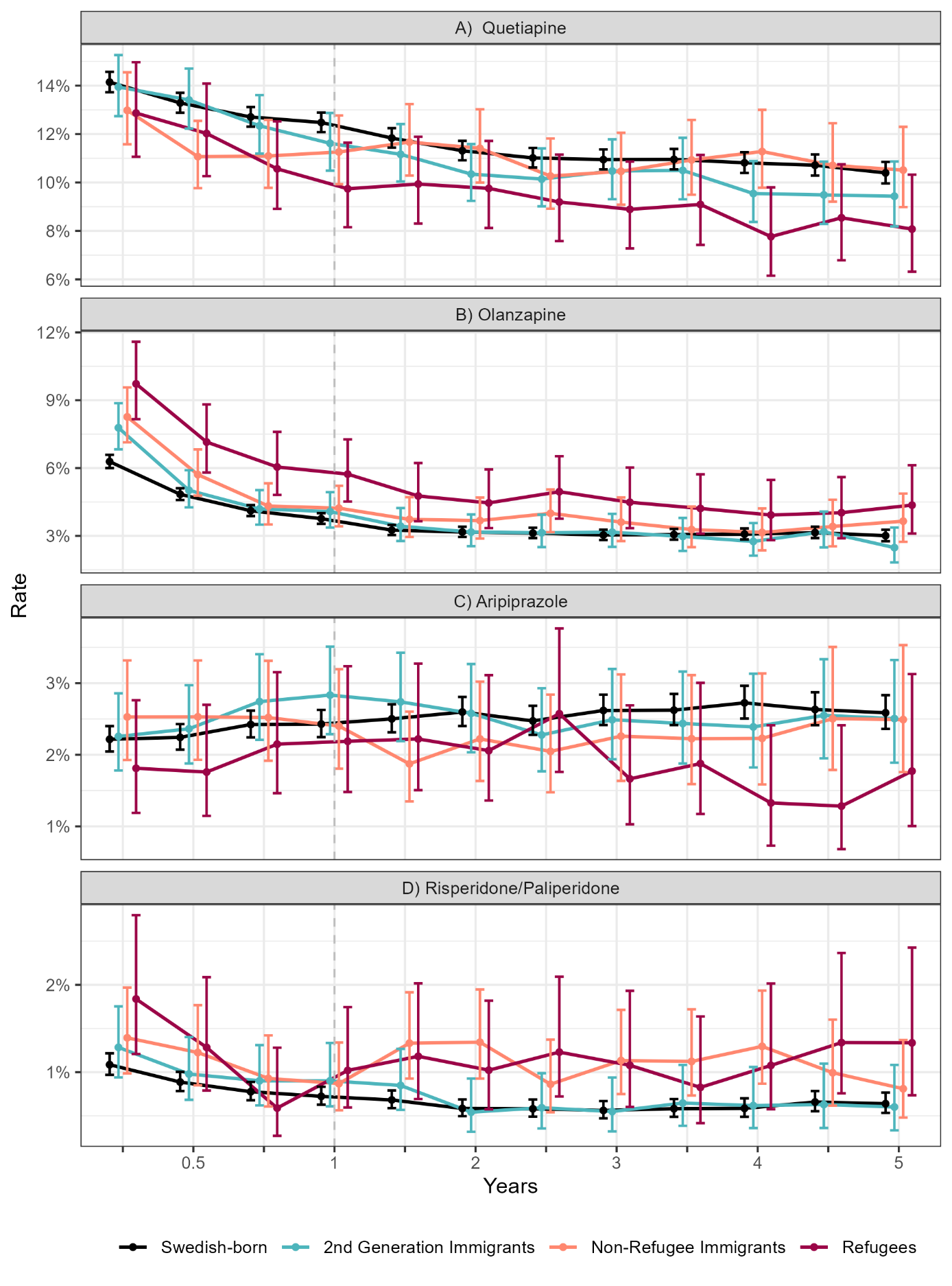


**Supplementary Figure 3.** Marginal means with corresponding 95% confidence intervals of estimated rates of antipsychotic use stratified by drug subclasses. Rates are adjusted for socio-demographic and comorbidity covariates and are pictured over the observation time of 5 years following diagnosis with bipolar disorder. Colored lines correspond to groups based on history of immigration. The group of second-generation immigrants was born in Sweden, however, to parents with personal migration history.


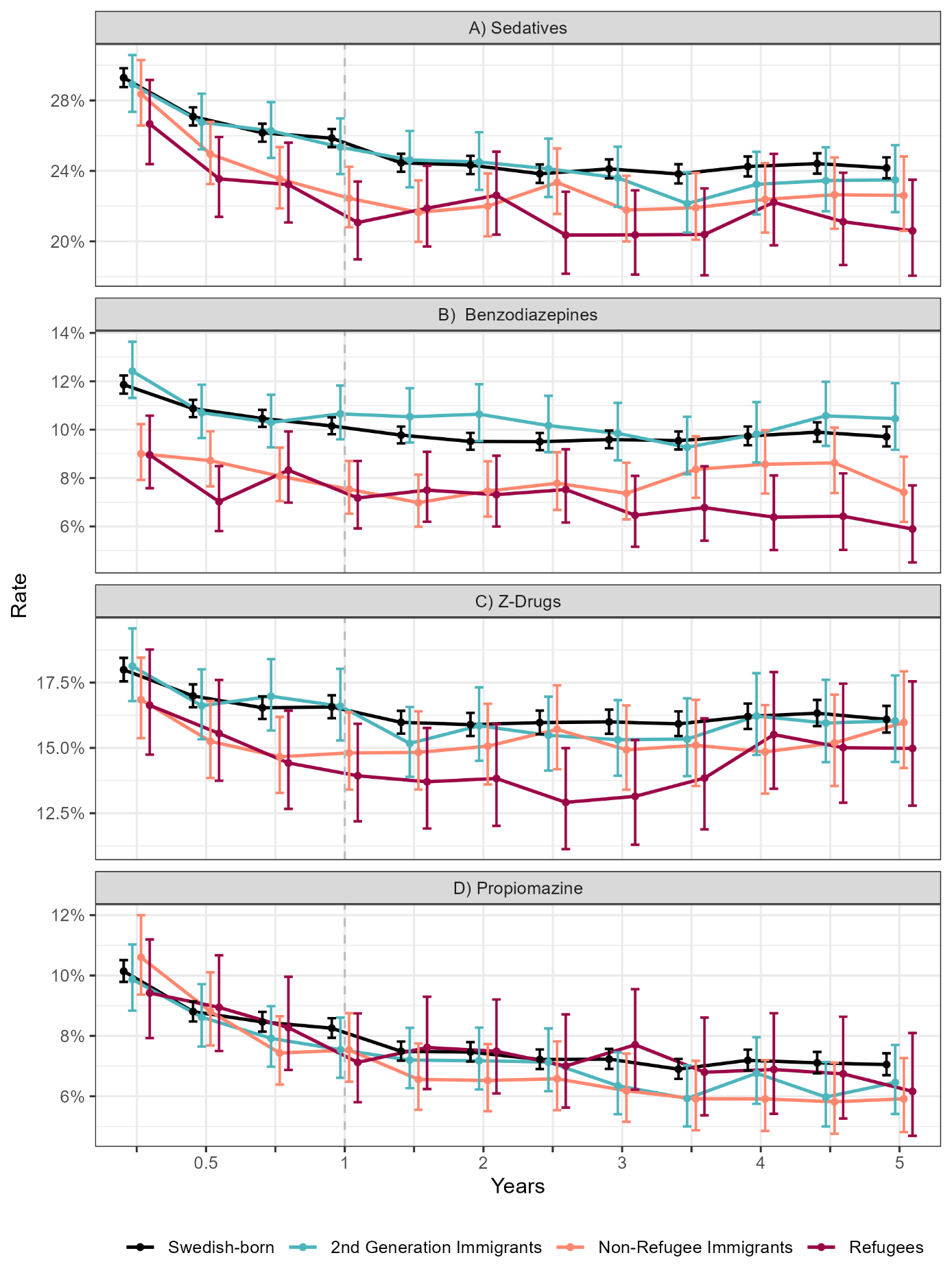


**Supplementary Figure 4.** Marginal means with corresponding 95% confidence intervals of estimated rates of sedative use and stratified drug subclasses. Rates are adjusted for socio-demographic and comorbidity covariates and are pictured over the observation time of 5 years following diagnosis with bipolar disorder. Colored lines correspond to groups based on history of immigration. The group of second-generation immigrants was born in Sweden, however, to parents with personal migration history.


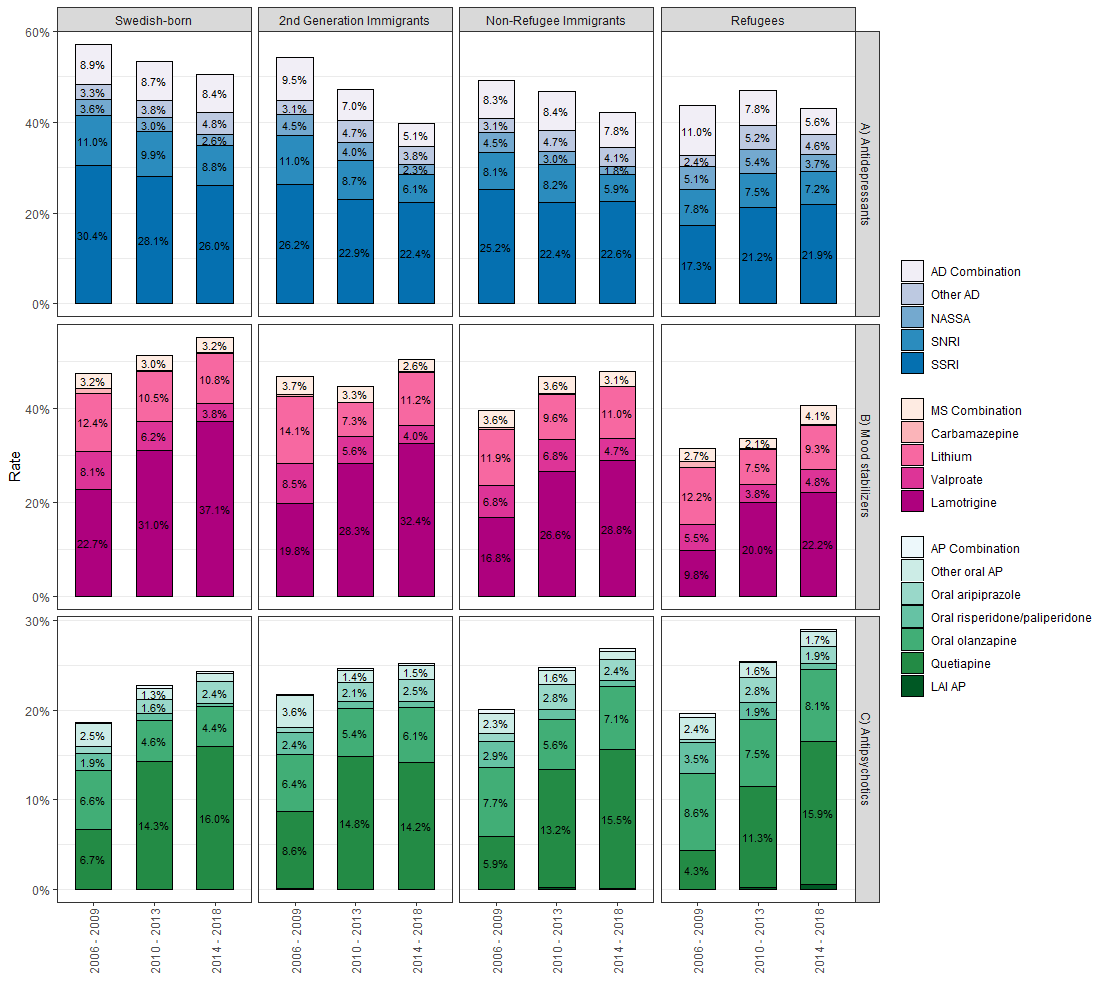


**Supplementary Figure 5.** Respectively for time-periods of diagnosis (2006-2009, 2010-2013, 2014-2018), bar plots depicting the composition of drug use by subclasses and specific drugs used 3 months after incident diagnosis of bipolar disorder. Drugs are grouped as A) antidepressants, B) mood-stabilizers, C) oral antipsychotics, and bar plots are shown respectively for groups based on immigration history. Crude rates of use are shown (without adjustment for socio-demographic and comorbidity covariates). The group of second-generation immigrants was born in Sweden, however, to parents with personal migration history.

Abbreviations: AD = antidepressant, AP = antipsychotic, LAI = long-acting injectable, MS = mood-stabilizer, NASSA = noradrenergic and specific serotonergic antidepressant (mirtazapine), SNRI = serotonin-noradrenaline-reuptake inhibitor, SSRI = selective serotonin-reuptake inhibitor


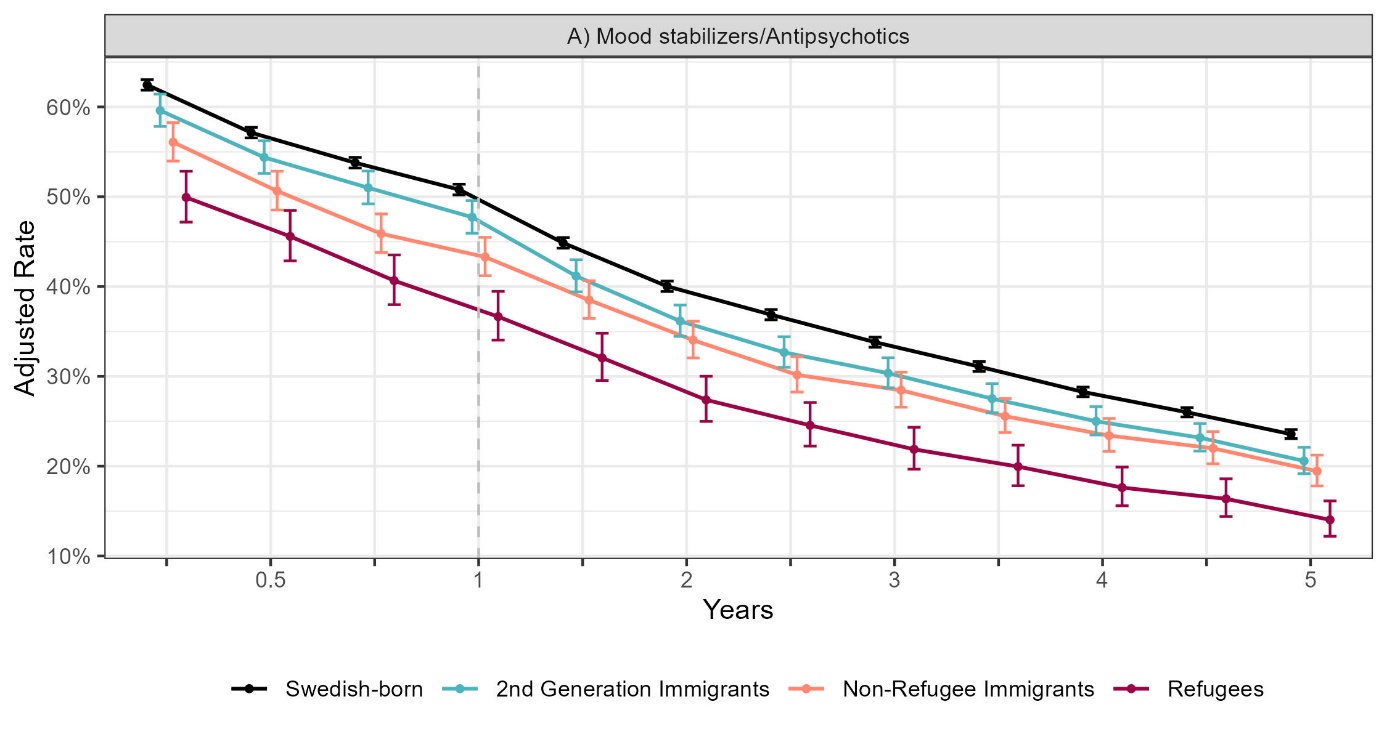


**Supplementary Figure 6.** Marginal means with corresponding 95% confidence intervals of estimated rates of adequate treatment, i.e., use of either mood-stabilizers or antipsychotics in any combination, including antidepressants. Rates are adjusted for socio-demographic and comorbidity covariates and are pictured over the observation time of 5 years following diagnosis with bipolar disorder. Colored lines correspond to groups based on history of immigration.


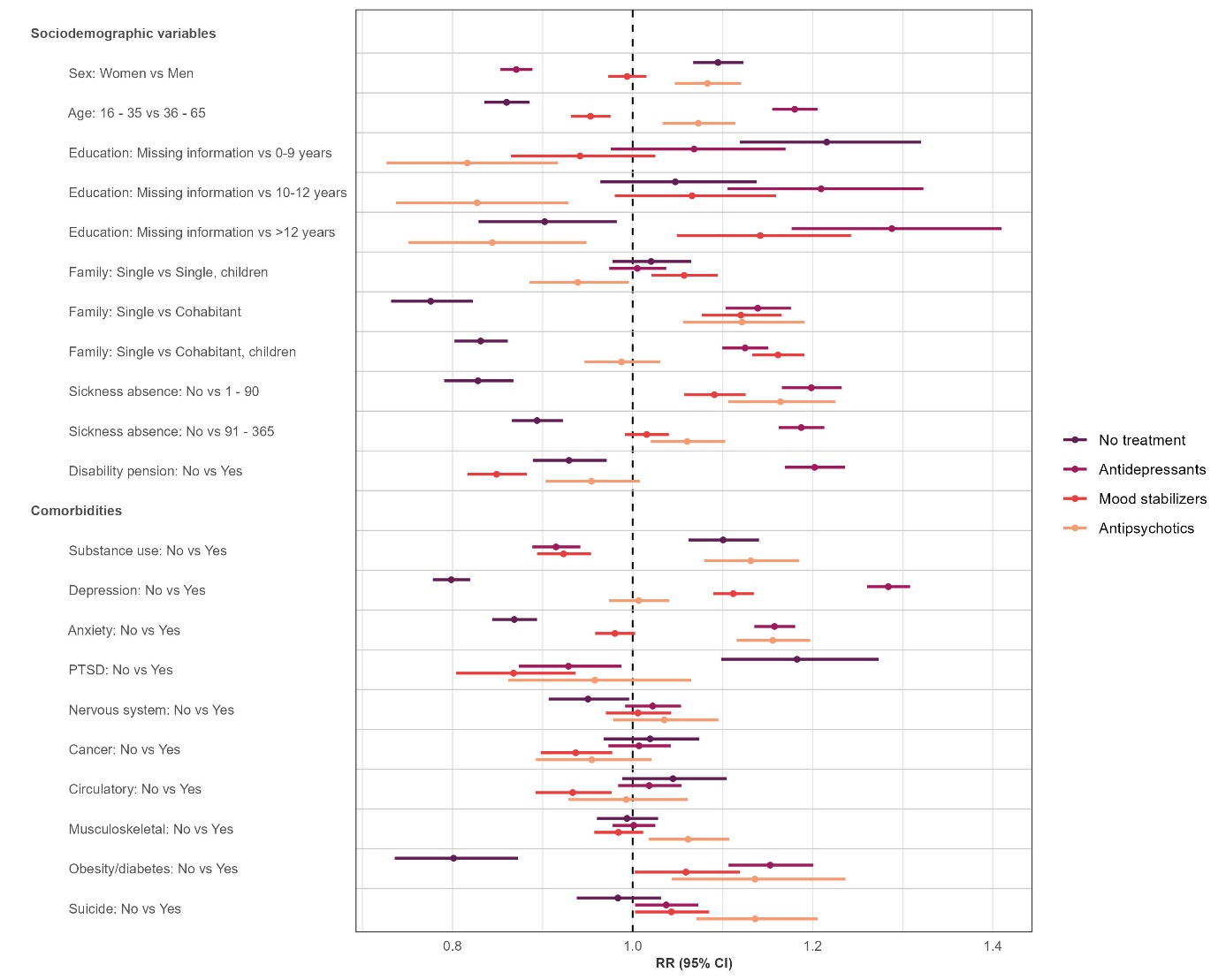


**Supplementary Figure 7.** The effects of each covariate, respectively adjusted for all other covariates, on the main outcomes, lack of adequate treatment, use of mood-stabilizers, antidepressants, and antipsychotics. were computed across the whole sample and are displayed as forest plots of relative risk (RR) with confidence intervals (CI). The group of second-generation immigrants was born in Sweden, however, to parents with personal migration history.
